# Organic-Inorganic Biocompatible Coatings for Temporary and Permanent Metal Implants

**DOI:** 10.3390/ijms252111623

**Published:** 2024-10-29

**Authors:** Lyudmila V. Parfenova, Zulfiya R. Galimshina, Evgeny V. Parfenov

**Affiliations:** 1 Institute of Petrochemistry and Catalysis, Ufa Federal Research Center, Russian Academy of Sciences, 450075 Ufa, Russia; lollipiip@mail.ru; 2 Department of Materials Science and Physics of Metals, Ufa University of Science and Technology, 450008 Ufa, Russia; evparfenov@mail.ru

**Keywords:** implants, plasma electrolytic oxidation, micro-arc oxidation, organic coating, biocompatibility

## Abstract

The general trend of increasing life expectancy will consistently drive the demand for orthopedic prostheses. In addition to the elderly, the younger population is also in urgent need of orthopedic devices, as bone fractures are a relatively common injury type; it is important to treat the patient quickly, painlessly, and eliminate further health complications. In the field of traumatology and orthopedics, metals and their alloys are currently the most commonly used materials. In this context, numerous scientists are engaged in the search for new implant materials and coatings. Among the various coating techniques, plasma electrolytic oxidation (PEO) (or micro-arc oxidation—MAO) occupy a distinct position. This method offers a cost-effective and environmentally friendly approach to modification of metal surfaces. PEO can effectively form porous, corrosion-resistant, and bioactive coatings on light alloys. The porous oxide surface structure welcomes organic molecules that can significantly enhance the corrosion resistance of the implant and improve the biological response of the body. The review considers the most crucial aspects of new combined PEO-organic coatings on metal implants, in terms of their potential for implantation, corrosion resistance, and biological activity in vitro and in vivo.

## 1. Introduction

In the demand for the restoration of the human musculoskeletal system, medical implants for osteosynthesis occupy a leading position. The service life of an implant in the body is a key factor in determining whether it is temporary or permanent. The implants that remain in the body between 3 and 12 months are considered to be temporary, while those that remain for 15 years or more are known as long-term or permanent [1,2]. A notable proportion of the devices installed are temporary implants, which are subsequently removed once the bone regrowth has occurred. It is not uncommon for long-term implants to require replacement within a year of the surgery due to complications associated with engraftment. This problem arises because an implant gets covered with nonspecific proteins when it enters the biological environment of a human body. The formation of such an adsorbed layer causes the foreign body response, which leads to the formation of a fibrous capsule that isolates the implant from the target tissue [3,4]. This results in the loss of the device. Moreover, the incorporation of antibacterial properties into implants is crucial for the elimination of the necessity for repeated surgical intervention, which may be associated with the potential for infection during the insertion of the device or postoperatively through the haematogenous spread of microorganisms from the source of infection [5].

**Figure 1:**
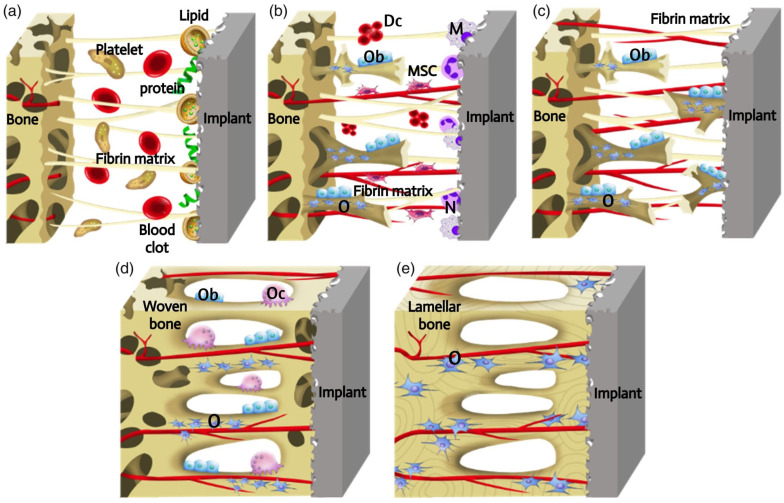
Schematic presentation of the process of implants osseointegration. The process of osseointegration includes the formation of the blood clot and fibrin matrix (**a**). Angiogenesis and woven bone formation (**b**). Distance osteogenesis and contact osteogenesis (**c**). Newborn woven bones fill up the gap, and bone is remodeling (**d**). Woven bones transform into lamellar bones (**e**). Dc, decomposed clot; M, macrophage; MSC, mesenchymal stem cell; N, neutrophil; O, osteocyte; Ob, osteoblast; Oc, osteoclast [6] (With permission from Ref. [6]; License Number: 5781780427577, License date: 4 May 2024).

The osseointegration is a dynamic process that involves a cascade of cellular and extracellular events (Figure 1) [6]. The introduction of the implant into the human body initiates an inflammatory response, during which proteins and lipids from the blood clot are adsorbed onto the implant surface. It is possible that these surface-coating proteins may act as a signal for cell migration and proliferation. Subsequently, platelets contribute to the formation of the fibrin matrix, which serves as a kind of “bridge” for the migration and attachment of cells. Following the placement of the implant, macrophages and neutrophils attach to it via a “bridge”, removing pathogens and necrotic tissue and breaking down the blood clot to make way for new blood vessels. This process occurs 2 to 3 days after the implantation. Following a period of 4 days, angiogenesis is initiated in the gap between the implant and the host bone, involving undifferentiated mesenchymal stem cells (MSCs), in order to produce new blood vessels. In the presence of growth factors and cytokines, MSCs undergo differentiation into osteoblasts, which are capable of producing extracellular matrix and forming immature bone tissue. However, MSCs can also differentiate into fibroblasts, which can stimulate the formation of a fibrous capsule on the implant surface and inhibit the process of bone ingrowth. The process of woven bone formation persists for a period of 1–2 weeks following implantation. Following a 2-week period, the space between the bone and the implant is filled with newly formed bone tissue. Subsequently, the osteogenesis process involves bone apposition and remodelling. During this process, osteoclasts resorb the newly formed bone, eliminating microfractures and optimising the lamellar bone surface. The processes of osteoclast and osteoblast activity are mutually reinforcing, with the former facilitating the transformation of the temporary woven bone into a more robust structure comprising parallel fibres. This then evolves into lamellar bone. This dynamic process occurs continuously for a period of at least 1 year, when the implant is fixed in the long term.

The review [7] highlights the importance of considering osteoimmunology in the process of osseointegration, i.e., how the properties of the implant surface (chemical composition, topography, hydrophilicity) influence the phenotypes of adherent cells, and how the interaction of immune cells with the device surface can alter their function.

Consequently, the materials employed in bone implantation must facilitate a specific interaction with the internal environment of the human body, and they must possess certain mechanical, physicochemical, and biological properties. The most crucial property among them is biocompatibility.

In this context, the world practice of providing biocompatibility and antibacterial properties of explants employs methods associated with the alteration of the architecture and composition of the surface layer, so that the devices comply with the properties of the bone tissue and cell membranes. This approach, which may be described as biomimetic, is widely developed. The modelling of surface properties is achieved through two distinct methodologies: physicochemical methods of coating formation and the application of an organic matrix containing molecules having different functions. The former approach involves the approximation of the phase composition and three-dimensional structure of the surface layer to the mineral components and morphology of the human bone, while the latter entails the use of an organic matrix containing fragments with varying functions [8]. Various methods are used to produce inorganic biocompatible coatings and modified surface layers: chemical treatment, electrophoretic deposition, plasma spray, chemical vapor deposition, oxygen plasma treatment, anodic oxidation, ion implantation, lithography, and plasma electrolytic oxidation [9]. These methods change the surface topography, chemical composition and, hence, functionality by the formation of oxide and hydroxide layers, the introduction of calcium phosphates, carbon, metals, functional particles, and nanoparticles [10].

**Figure 2:**
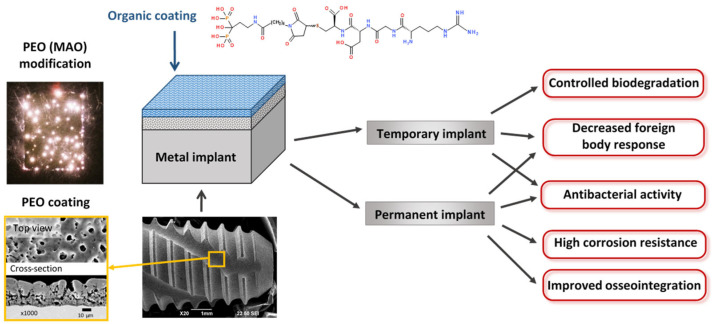
Typical dental implant with PEO coating. Surface modification of temporary and permanent implants.

Among these various surface treatments, plasma electrolytic oxidation (PEO) (or micro-arc oxidation—MAO) appears to be the most promising. Plasma electrolytic oxidation is a progression of anodizing into higher voltages. Advanced PEO units use bipolar voltage, typically from 400 to 500 V for the positive pulse and from 20 to 100 V for the negative pulse, applied at relatively high frequencies ranging from 50 to 2000 Hz [11]. To obtain biocompatible coatings, alkaline, phosphate, silicate electrolytes are used with additions of calcium acetates or citrates [12]. The coatings produced by the PEO are based on stable metal oxides that are reliably bonded to the surface due to the mechanism of coating formation, which includes electrochemical oxidation and multiple remelting events of the surface under the action of microdischarges that appear within the oxide layer at the high voltages applied. This process allows the production of coatings 5 to 100 µm thick, with adjustable porosity, and pore sizes ranging from 0.1 to 10 µm. A typical implant with PEO coating is shown in Figure 2. The developed pore network exhibits a fractal structure, with pores becoming increasingly larger towards the surface [11]. This morphology facilitates a gradual transition in elastic modulus from the metal implant to the surrounding bone tissue, thereby enhancing biomechanical compatibility. This is achieved through the biomimetic (at the physicochemical level) surface, which closely resembles the natural bone tissue. The developed surface of the PEO coating facilitates the adhesion of cells to the implant surface [13]. The application of pulses of alternating polarity during the PEO allows the inclusion of anions and cations of the electrolyte into the coating composition, thereby facilitating the formation of Ca- and P-containing bioactive crystalline phases included in the coating composition: hydroxyapatite (HA), tricalcium phosphate, tetracalcium phosphate, perovskite. The adhesion of PEO HA-containing coatings is demonstrably superior to that of other methods [14,15]. The application of duplex PEO in conjunction with electrophoretic deposition enables the incorporation of nanoparticles of hydroxyapatite, silver, and other materials into the composition of the coating [16,17]. Consequently, research into the design of biocompatible coatings on metallic implants using PEO is progressing at a high rate [18].

The modelling of surface properties by the application of an organic matrix containing functional fragments can regulate the biological properties of the surface [19]. Inorganic coatings discussed above are typically distinguished by their high adhesion, developed surface, and mechanical properties that closely resemble those of a bone. However, due to their inorganic composition, they exhibit limited bioactivity. The bioactivity of inorganic coatings can be significantly enhanced and fine-tuned by the incorporation of an organic matrix, which can either mask the implant or actively interact with the cells. A number of methodologies have been developed for the design of biocompatible surfaces for implants [4,20]. Firstly, the surface can be treated to prevent the formation of a non-specific protein layer, rendering the device “invisible” to body cells (so-called non-fouling coatings). Polysaccharides, self-organising monolayers, polyethylene glycols, polyacrylates, and other materials are employed in the formation of such surfaces. Secondly, it is possible to apply compounds to the surface of the implant that will provide an appropriate signal between the implant surface and cell surface receptors (proteins, protein fragments, growth factors, etc.). The approach that combines both non-fouling and biologically active layers appears to be the most promising, as the resulting coating will enhance the quality of implants by reducing or completely blocking non-specific adsorption of proteins and forming specific binding sites for certain cell types. Furthermore, organic coatings, in addition to their biocompatibility, can fulfil the function of controlling the corrosion rate of active metals (e.g., Mg, Zn) used for the fabrication of temporary implants.

This strategy is summarized in Figure 2. The metal implant either of a temporary, or of a permanent type, is coated by the PEO modification following the biomimetic approach. Further, the top organic coating provides specific chemical and biological properties to the surface.

Therefore, the objective of this review is to examine the potential of combined coatings based on organic compounds and porous inorganic coatings obtained by the PEO (MAO) method for modelling the surface properties of temporary and permanent implants.

## 2. Temporary Implants Based on Biodegradable Metals

**Figure 3:**
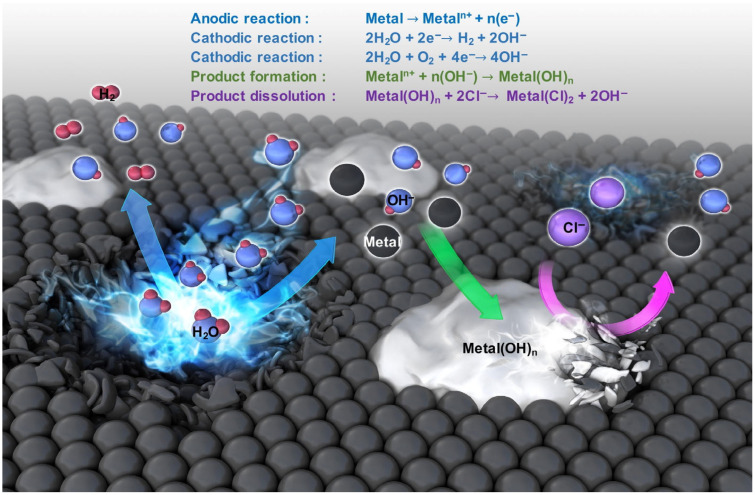
Mechanism of metal biodegradation [22]. (With permission from Ref. [22]; License Number: 5781780723366, License date: 4 May 2024).

In recent decades, there has been an active search for materials that exhibit high biocompatibility, mechanical strength, controllable corrosion resistance, and low toxicity, with the aim of developing temporary biodegradable implants. Among metals, biodegradable magnesium, iron, zinc, and their alloys are of particular interest in this field. The application of the aforementioned metals and their alloys is constrained due to the generation of corrosion products that are released at the implanted site (Figure 3). These corrosion products can potentially damage the body, necessitating re-surgery [21,22].

Among metal-based biodegradable materials, magnesium and its alloys are most widely recognized for the temporary implant applications due to their appropriate mechanical strength and biocompatibility [23]. Nevertheless, their low corrosion resistance represents a significant limitation to the wide use of magnesium-based implants. Therefore, there is a high demand to find anti-corrosion coatings that would control biodegradation kinetics and also possess developed surface and good wettability. Currently, various methods of coating formation are used to protect Mg and its alloys in the body fluids (Table 1). The PEO (MAO) method is currently under active development. Nevertheless, the oxide layer produced by PEO can be brittle and susceptible to destruction, and it may accelerate local corrosion when implanted in the body [24,25]. In this regard, the search for a suitable organic top coating is underway; this coating should fill the micropores and microcracks of the PEO-coated biodegradable metal, and, ideally, should provide high biocompatibility [10,26]. Various types of organic molecules that address this issue, would be discussed below.

### 2.1. Synthetic Biocompatible Polymers

**Figure 4:**
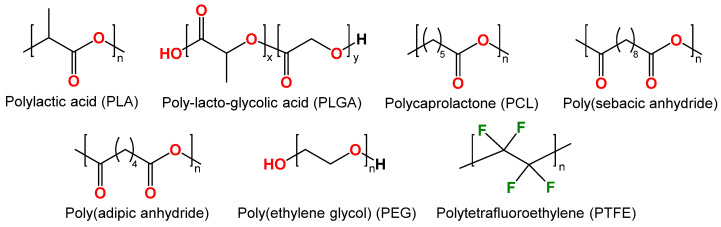
Structure of synthetic biodegradable polymers: polylactic acid (PLA), poly(lactic-co-glycolic) acid (PLGA), polycaprolactone (PCL), poly(sebacic anhydride), poly(adipic anhydride), poly(ethylene glycol) (PEG) and polytetrafluoroethylene (PTFE).

In recent decades, synthetic polymers such as polylactic acid (PLA), poly(lactic-co-glycolic) acid (PLGA), polycaprolactone (PCL), and others have been successfully employed as anti-corrosion coatings (Figure 4). These polymers are biodegradable, biocompatible, thermoplastic, and commercially available [44,45].

**Polylactic acid (PLA).** Polylactic acid (PLA) (Figure 4) belongs to aliphatic polyesters due to the ester bonds connecting the monomer units of lactic acid. PLA has gained a key role in the biomedical field for a wide range of applications, including suture threads, screws for bone fixation, and drug delivery devices [46]. PLA gained its popularity due to production from renewable resources, which is economically viable. Its monomer (lactic acid) is typically derived from fermented plant starch, such as corn, sugarcane, or sugar beet. Lactic acid polymerization can be achieved through either microbial fermentation or chemical polymerization [47].

For example, PEO composite coatings comprising polymeric PLA were prepared on biomedical Mg-1.21Li-1.12Ca-1.0Y alloy [27]. The PEO of the Mg-1.21Li-1.12Ca-1.0Y alloy was conducted in a silicate electrolyte containing NaOH, Na_2_SiO_3_, NaB_4_O_7_, Na_3_C_6_H_5_O_7_, and phytic acid. PLA coatings were prepared on the PEO-modified surface by dipping metal samples in a solution of 1% PLA in dichloromethane under ultrasonic vibration. The corrosion resistance of the Mg-1.21Li-1.12Ca-1.0Y alloy was found to be significantly enhanced by the application of a PEO/PLA coating. The hydrogen release rate in Hank’s solution of PEO-treated Mg-1.21Li-1.12Ca-1.0Y alloy exhibited a gradual increase, eventually exceeding that of the uncoated alloy. In contrast, the hydrogen release rate of PEO/PLA remained at a lower level. The PEO/PLA coating was observed to alter the pH of the solution to a range of 7.5–7.8 within 3 days, which may prevent the tissue from becoming alkalinized on the magnesium surface.

Biodegradable PLA-based composites reinforced with high-strength AZ31 magnesium alloy wires (MAWs) are manufactured for the purpose of bone fixation [48]. The materials exhibit remarkable strength due to the unidirectional reinforcing effect exerted by MAWs. The durability of the composite can be enhanced by the application of composite coatings. In order to achieve this objective, MAW was prepared from AZ31 (96% Mg, 3% Al, and 1% Zn by weight) through a series of processes, including continuous melting, casting, hot extrusion, wet drawing, and annealing. PEO coating was applied to MAW from a solution consisting of sodium silicate, sodium phosphate, sodium hydroxide, and KZrF_6_. The PEO coating enhanced the interfacial bonding between the MAWs and the PLA, thereby increasing the tensile and flexural strength of the resulting composites. Furthermore, it has been demonstrated that the rate of PLA degradation is reduced following the introduction of PEO-treated MAWs.

A composite coating on biodegradable AZ31 alloy was created by combining a PEO coating with PLA [28]. Porous PEO coatings on AZ31 alloy were prepared in electrolytes containing Na_2_SiO_3_·9H_2_O, NaOH, and NaF. Subsequently, the PEO-coated samples were immersed in a 6% solution of PLA in dichloromethane. It was indicated that the PEO/PLA composite coating markedly enhanced the corrosion resistance of the AZ31 alloy. The corrosion potential exhibited a shift from −1.663 V to a more positive value of −1.317 V, accompanied by a six-order-of-magnitude decrease in corrosion current density. The formation rate of Mg^2+^ ions, hydrogen release, and the change in solution pH value caused by degradation were significantly reduced. Moreover, the PEO coating played a crucial role in maintaining the integrity of the implant during long-term use. The hemolysis test demonstrated that the PEO/PLA coating conferred a low hemolysis rate of (0.806 ± 0.771)% on the AZ31 alloy, which is well below the safe value of 5%. In the cytocompatibility test, the PEO/PLA composite coating exhibited superior biocompatibility compared to the pure AZ31 alloy and PEO coating. The PEO/PLA coating demonstrated significantly enhanced adhesion and proliferation of MC3T3-E1 cells, with a four-fold increase in cell number. This indicates that the composite coating is suitable for biomedical applications.

Porous PEO/PLA composite coatings were applied to an extruded Mg-1Li-1Ca alloy (1.26 wt% Li, 0.95 wt% Ca, Mg balance) [29]. The PEO coating was prepared in the electrolyte comprising sodium hydroxide and phytic acid. A 5 wt% solution of PLA in dichloromethane was used to apply a PLA coating to a PEO/Mg-1Li-1Ca. The corrosion resistance evaluation demonstrated that degradation predominantly occurs at the interface between the PEO coating and the organic substrate. The accumulation of corrosion products and hydrogen bubbles caused swelling and bubble formation on the PLA coating due to the higher rate of water penetration into the polymer coating than its degradation rate. The acidic and alkaline products resulting from the decomposition of PLA and the corrosion of magnesium, respectively, were found to neutralize each other, thereby maintaining a stable pH value in Hanks’ solution (HBSS). The application of PEO/PLA coating to magnesium alloys demonstrated significant enhancement of their cytocompatibility and blood compatibility, also providing an appropriate solution pH.

A systematic study was conducted to investigate the effect of applying PLA polymer on AZ31 alloy modified with PEO [30]. The applied coating was optimized using Taguchi design of experiment, which is a method designed to develop systems for more reliable and stable product improvement under uncontrolled conditions [49]. The impact of processing parameters, including the number of layers, sample extraction rate, and polymer concentration, on mass gain, thickness, and roughness characteristics, was assessed. The adhesion and corrosion properties of the coatings were also evaluated. The relationship between the parameters of the PLA solution and the final performance of the coating differs between the two systems under study, AZ31 and AZ31/PEO. The study demonstrated that the number of layers and the extraction rate are not critical parameters for the AZ31/PLA system. However, for the AZ31/PEO/PLA system, increasing the number of layers or decreasing the rate results in an increase in sample mass gain. An increase in the concentration of the polymer results in a corresponding increase in the thickness of the PLA layer. The results indicate that the adhesion strength of PLA coatings to the Mg substrate is influenced by three key factors: (i) the number of layers, (ii) the solution concentration, and (iii) the extraction rate used in the dip coating process. The maximum adhesion strength value obtained using PLA coatings on AZ31 was 8.1 MPa, which was 50% higher than the maximum value obtained using the PEO interlayer. The adhesion properties of PLA on AZ31/PEO substrates were found to be inferior to those of PLA on bare magnesium substrates. This was attributed to the cohesive failure of the PEO coating. The elevated surface roughness of the PEO/PLA system is attributed to the absorption of moisture by the PEO pores, which is a particularly destructive phenomenon for metal-to-polymer bonding. In terms of corrosion resistance, the PEO layer reduces the corrosion current density by two orders of magnitude compared to the value of the pure substrate. Furthermore, the double layer of PEO and PLA provides an additional improvement. The study indicates that the limitations observed in the AZ31/PEO system are attributable to the brittle and defective nature of the oxides formed during PEO processing. The PLA layer serves to mitigate the adverse effects of these properties, as it fills the pores and seals the cracks of the PEO layer.

The mechanical behavior, corrosion mechanisms, and cytocompatibility at the interface of magnesium wire-reinforced PLA composites were investigated in [50]. The surface modification of magnesium wires by PEO resulted in an increase in the interface shear strength from 10.9 MPa to 26.3 MPa. However, this decreased to 8 MPa and 13.6 MPa in Mg/PLA and PEO-Mg/PLA composites, respectively, following 42 days of in vitro degradation. In general, the PEO surface modification enhanced the interface characteristics between the magnesium wires and the PLA matrix in both the original and damaged states, thereby protecting the magnesium wires from corrosion. In vitro studies on MC3T3-E1 osteoblasts demonstrated that the composite cross-sections exhibited good biocompatibility, regardless of the presence of the PEO layer. The cells migrated into the PLA regions and formed a monolayer, but avoided the surface of the Mg wires, indicating that Mg/PLA composites should be fabricated in a manner that ensures complete encapsulation of the Mg reinforcement within the PLA matrix.

**Poly (ε-caprolactone).** Poly (ε-caprolactone) (PCL) (Figure 4) is a synthetic biodegradable aliphatic polyester that has attracted the attention of researchers especially in biomedical fields related to controlled-release drug delivery systems, absorbable surgical sutures, nerve conductors, and three-dimensional scaffolds for tissue engineering applications [51]. In contrast to PLA, PCL has a relatively low melting point of 60 °C (140 °F) and is subject to slower biodegradation. This material is well-suited for low- to no-stress applications, and it has been approved by the FDA for use in implantable medical devices.

**Figure 5:**
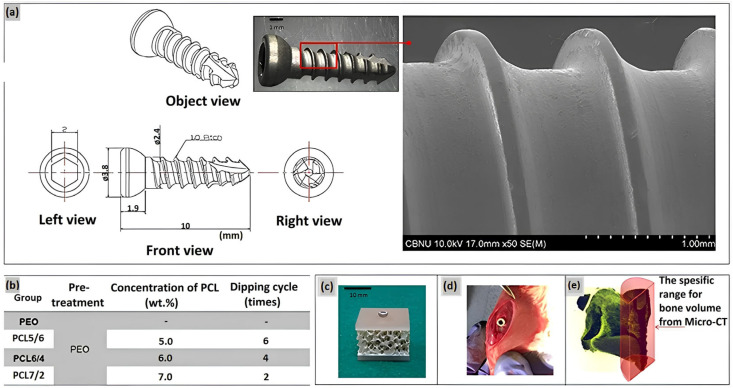
(**a**) Design of the screw and surface morphology; (**b**) Conditions of coating the PEO Mg screw by PCL, (**c**) artificial bone plate model for the immersion test, (**d**) in vivo model in a rat tibia, and (**e**) bone volume measurement range [32].

A duplex coating on magnesium has been developed, comprising an inner PEO coating layer and a top layer of a degradable PCL polymer [31]. An alkaline electrolyte based on silicate and fluoride was employed in the treatment of magnesium by the PEO method. A uniform polycaprolactone (PCL) coating layer was formed on Mg with PEO layer by immersing the material in a PCL solution at two concentrations: 7 wt% and <5 wt%. The corrosion resistance of PEO-PCL duplex-coated Mg was evaluated using a potentiodynamic polarization study and an immersion test in Hanks’ solution, which imitates the composition of a human blood plasma. The results demonstrated that Mg-PEO enhanced the corrosion resistance of the original Mg by a factor of 106, and Mg-PEO treated at a low PCL concentration by a factor of 139. Furthermore, Mg-PEO at a PCL concentration of 7 wt% increased the corrosion resistance of the original Mg by a factor of 28,000. A series of studies have demonstrated that the pH of HBSS, in which pure Mg and Mg-PEO samples were immersed, reached a value of 8.32 and 8.15, respectively. The observed increase in pH levels provides compelling evidence that pure magnesium is irreversibly corroded by HBSS treatment, and that PEO treatment is unable to provide corrosion protection. In contrary, the application of a layer of PCL at a concentration of 5 wt% on Mg-PEO results in a pH value of 7.5–7.6, which is relatively low. This indicates that the polymer coating has the ability to reduce the corrosion rate and provide corrosion protection in a human blood plasma. In another research of the PEO surface treatment of Mg, the homogeneity of the coating was modified by varying the concentration of PCL and the number of layers on the Mg-PEO surface (Figure 5) [32]. The PEO electrolyte comprised NaOH, Na_3_PO_4_, and glycerol. The application of PCL coatings was conducted with varying concentrations (5–7 wt%) in a total of two to six coats. Consequently, PEO-modified Mg samples with PCL coatings of 5 wt% in six layers (PEO/PCL5/6), 6 wt% in four layers (PEO/PCL6/4), and 7 wt% in two layers (PEO/PCL7/2) were obtained. All of the obtained samples were utilized as coatings on screws manufactured from magnesium. Upon examination of the coatings, it was demonstrated that the PEO/PCL5/6 and PEO/PCL7/2 coatings exhibited inhomogeneous structure, which resulted in the presence of oxides and gases between the Mg layer and PCL. This phenomenon was attributed to internal corrosion of magnesium and flaking of the coating. The PEO/PCL6/4 coating exhibited high corrosion resistance due to low thread wear. In vivo analyses demonstrated that the PCL-coated screw induced the formation of denser and thicker bone around the rat femur compared to Mg and Mg-PEO.

A PEO coating was fabricated on an AZ31 alloy in an electrolyte solution containing Na_2_SiO_3_·9H_2_O, KOH, and KF·2H_2_O [33]. AZ31-PEO was coated with PCL via immersion in a dichloromethane solution with a concentration of 5 wt%. The PEO/PCL coating was subsequently treated with polydopamine (PDAM) via the immersion method. The evaluation of corrosion parameters of AZ31-PEO, AZ31-PEO/PCL, and AZ31-PEO/PCL/PDAM, as well as the AZ31 alloy, demonstrated a significant increase in corrosion resistance, with an order of magnitude increase observed for AZ31-PEO and a five-fold increase observed for AZ31-PEO/PCL and AZ31-PEO/PCL/PDAM compared to AZ31 alloy. In a simulated body fluid (SBF), the AZ31 and AZ31-PEO samples exhibited localized alkalinization, accompanied by a notable increase in the pH value of the SBF solution, as a result of their Mg corrosion. The pH values of the simulated body fluid remained largely unchanged for both composite samples containing polymer and polydopamine. Cytotoxicity analysis revealed that cell viability on AZ31-PEO was marginally reduced in comparison to AZ31, yet remained above 70%, indicating that the material exhibited no overtly cytotoxic properties. The AZ31-PEO/PCL and AZ31-PEO/PCL/PDAM coatings were found to be non-cytotoxic. A direct incubation of the MC3T3-E1 cells on different surfaces revealed that the cells exhibited higher adhesion and faster proliferation on the AZ31-coated PEO/PCL and PEO/PCL/PDAM surfaces compared to the AZ31 and AZ31-PEO surfaces. The in vitro antibacterial efficacy evaluation against *S. aureus* and *E. coli* demonstrated that AZ31-PEO/PCL and AZ31-PEO/PCL/PDAM exhibited no antibacterial activity. In contrast, the AZ31 alloy demonstrated strong antibacterial ability due to the rapid increase in pH value caused by Mg corrosion. Additionally, the PEO-coated sample showed the retention of its antibacterial efficacy, accompanied by a slight elevation in the pH value. The immobilization of polyhexamethyleneguanidine (PHMB) on the surface of the AZ31-PEO/PCL/PDAM coating resulted in the enhancement of its antibacterial efficacy.

**Figure 6:**
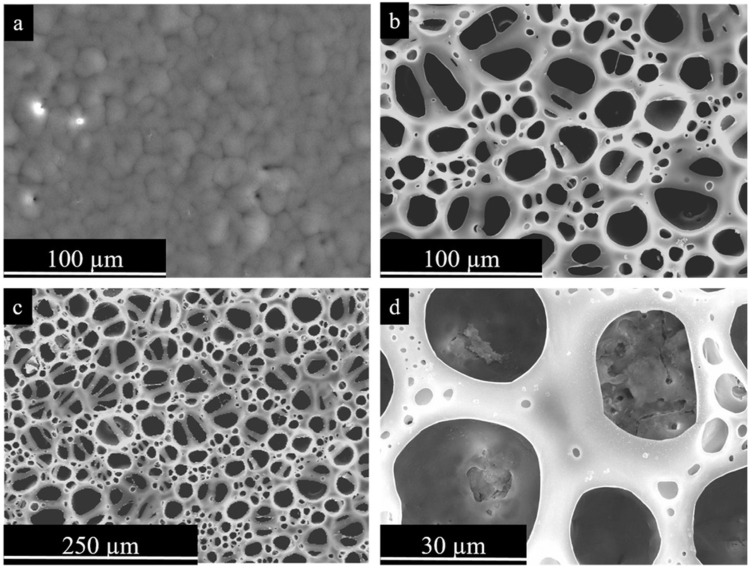
Secondary electron plan view micrographs of PEO-PCL coatings. (**a**) Mg-PEO-PCL without porosity; (**b**–**d**) Mg-PEO-PCL-BF at different magnifications [34]. (With permission from Ref. [34]; License Number: 501904643, License date: 8 May 2024).

The Mg/PEO/PCL/BF coating system is described in Ref. [34] (Figure 6). In this work, the so-called breath figures (BF) approach was employed in order to generate a porous polymer structure on the magnesium surface. The BF technique involves the formation of a porous polymer layer at a relative humidity (RH) of greater than 90% through the use of a polymer solution in a volatile solvent. As the solvent evaporates, the temperature at the interface drops, leading to condensation of water droplet vapor on the sample surface. This results in the polymer solidifying around the droplets, forming a porous structure [52]. The resulting coatings, when combined with a magnesium oxide porous surface, demonstrated enhanced adhesion and proliferation of premiblast cells in comparison to the control.

A hierarchical hybrid coating (HHC) was developed, comprising a PEO ceramic oxide layer and two biodegradable PCL polymer layers with ciprofloxacin (CIP) on the Mg3Zn0.4Ca alloy [35]. This coating was designed to provide a controlled degradation rate and functionality, creating a favorable porous structure on the surface for improved cell adhesion. The BF method was employed to fabricate a porous PCL polymer layer on top of Mg that had been subjected to PEO treatment. The HHC system with CIP drug provides an overall higher and more stable corrosion protection, which is increased by orders of magnitude due to the deposition of insoluble chelates that prevent aggressive media agents from accessing the HHC layers of PCL and PEO. This reduces the rate of Mg degradation. Local current density distribution maps demonstrated that ciprofloxacin-loaded HHC-coated Mg3Zn0.4Ca alloy exhibited an inhibitory effect approximately one order of magnitude greater than that of Mg3Zn0.4Ca and HHC-coated PCL and PEO alloy. The mechanism of active corrosion protection involves an initial, transient acceleration of Mg corrosion due to the formation of a complex of released Mg^2+^ ions, followed by the coating defect-blocking action of precipitated insoluble chelates of Mg^2+^ and Ca^2+^ cations with the continuously released zwitterionic form of ciprofloxacin. The results demonstrated that the HHC with CIP facilitated a gradual drug release over a period of 240 h. As a result, a multi-level protection system was developed for magnesium alloy by sealing the pores of the PEO coating with a polymer layer and inhibiting the corrosion process with CIP, which demonstrated an inhibitory effect of up to 74%. The corrosion inhibition effect of HHC and eluted drug is attributed to the formation of insoluble CIP-Me (Mg/Ca) chelates, which eliminate defects in HHC and prevent access of corrosive agents. Further, the system Mg/PEO/PCL/BF was functionalized with paracetamol (PAR) and ciprofloxacin(CIP) and characterized in terms of biological responses to drug release kinetics [36]. In vitro assays with cell lines required for cardiovascular implants and bone prostheses (endothelial cells and pre-osteoblasts) demonstrated that HHC with CIP facilitated proliferation and cell viability. Additionally, delayed drug release was observed. However, comprehensive system analyses demonstrated that the PCL layer formed bubbles that locally accelerated corrosion in the microenvironment of the slits, thereby affecting cell viability. A reduction in cytocompatibility was observed at the coating/substrate interface, which is likely due to an increase in the local pH of the material. This increase, which was up to 0.2 pH units per volume compared to the pre-culture medium, was caused by corrosion of the magnesium alloy.

The thickness of the polymer coating and the number of layers applied to the device can have varying effects on both corrosion resistance and the biological response. For instance, PCL coatings were successfully applied to PEO-treated ZM21 magnesium alloys. The PEO process was conducted in an electrolyte solution comprising Na_2_SiO_3_·9H_2_O, KOH, and Na_2_B_4_O_7_·10H_2_O [37]. The PCL coatings on magnesium alloys were prepared by immersing ZM21 in a 3% (wt./vol.) solution of chloroform and dichloromethane for 1, 5, and 10 s, resulting in samples designated ZM21/PEO/PCL1, ZM21/PEO/PCL5, and ZM21/PEO/PCL10, respectively. The application of PCL to PEO-coated samples demonstrated a significant improvement in corrosion resistance, with a 10^3^-fold increase compared to the PEO-treated specimen. The ZM21/PEO/PCL5 sample exhibited superior corrosion resistance compared to other samples. This suggests that the immersion time, in conjunction with an appropriate polymer thickness and morphology, plays a significant role in determining the corrosion resistance. However, the ZM21/PEO/PCL10 specimen with greater thickness exhibits reduced corrosion resistance due to its larger pores. Additionally, the adhesion of PCL on the PEO surface of ZM21 alloy was found to be low for this sample. The ZM21/PEO/PCL5 material exhibited the highest biological activity in the in vitro cytotoxicity testing, with a significantly lower number of dead cells even at 100% extract concentration. Furthermore, the sample demonstrated superior cell growth and proliferation in the direct contact assay.

Therefore, synthetic polymers in combination with the PEO coating on Mg alloys can control the biodegradation rate and provide biocompatibility.

### 2.2. Natural Polymers for Temporary Implants

**Figure 7:**
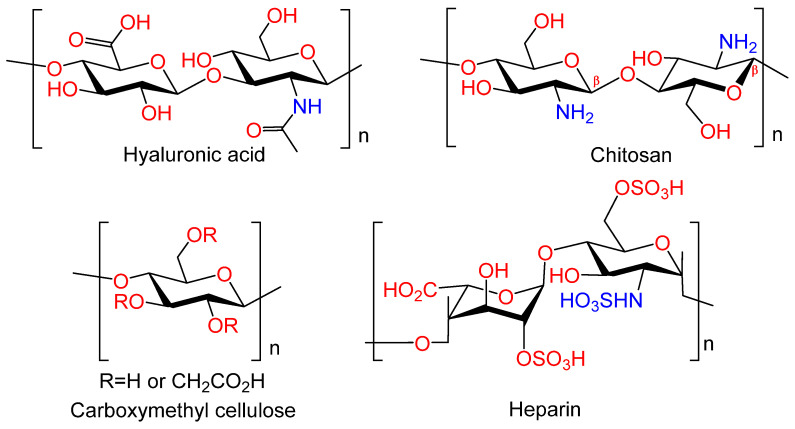
Natural polymers.

Polysaccharides, naturally occurring carbohydrates (Figure 7), exhibit low toxicity, high biocompatibility, and in vivo biodegradation ability. These properties render them as ideal candidates for use in a variety of biomedical devices, implants, and tissue engineering applications. Furthermore, numerous polysaccharides have been demonstrated to exhibit a multitude of additional biological actions, including antimicrobial, anticoagulant, antioxidant, immunomodulatory, hemostatic, and anti-inflammatory effects [53]. These properties may potentially confer additional therapeutic benefits to the metal implants.

**Hyaluronic acid.** Hyaluronic acid (HYA) occupies a unique position among polysaccharides, serving as a crucial component of the intermolecular matrix of all connective tissues. This high biocompatibility is a defining feature of HYA. It is a viscoelastic, linear regular copolymer of D-glucuronic acid and N-acetyl-D-glucosamine, which is synthesized by chondro- and osteoblasts; it is found in the extracellular matrix (ECM) in complex with proteins [54]. HYA is involved in the majority of biological processes, including cell motility, proliferation, tissue organization, wound healing, angiogenesis, and morphogenesis. HYA plays an important role in the development, growth, and reconstruction of the skeleton [55]. The biological activity of HYA depends on its molecular weight. For example, low molecular weight HA participates in the differentiation of osteoclasts [56]. It has been demonstrated that osteoblast differentiation is also sensitive to hyaluronan levels. Leukemia cell inhibitory factor (LIF) has been shown to suppress osteoblast development by stimulating the synthesis of high molecular weight HYA [57]. Due to their unique properties, hydrogels based on HYA, including chemically modified ones, have become a prevalent material in the field of tissue engineering. Their primary applications include bone tissue engineering and regeneration [53,58,59]. Consequently, HYA and its derivatives can contribute to the realization of the biomimetic approach by regulating the biological activity of the coating due to HYA high affinity to biological targets.

**Figure 8:**
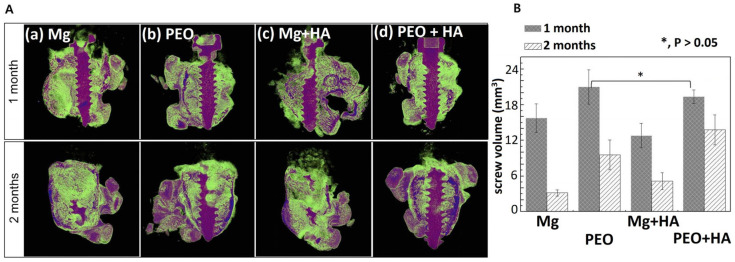
In vivo study of Mg screws with PEO/HYA/CMC coating. Micro-CT image of rat femur with screw (**A**) and screw volume by micro-CT (**B**) after implantation for 1 and 2 months [25]. (With permission from Ref. [25]; License Number: 501904646, License date: 8 May 2024).

Hyaluronic acid and carboxymethylcellulose (CMC) were applied to PEO-modified magnesium to obtain coatings that act as a film-type protective barrier to improve bonding function [25]. The corrosion test demonstrated that the initial corrosion resistance of the obtained coatings was enhanced by a factor of two in comparison to the original magnesium, due to the HYA coating. Additionally, the scratch test revealed that HA and CMC on PEO-Mg reacted with Ca^2+^ ions in SBF, which facilitated rapid self-healing. When Mg-HYA without PEO treatment was implanted into rat femur bone, the HYA coating peeled off, resulting in localized and persistent corrosion. In contrast, Mg/PEO/HYA demonstrated that HYA rapidly binds to Ca^2+^ ions and apatite in body fluids, thereby facilitating the healing of the outer layer of the damaged area. Meanwhile, magnesium oxide grows from the inner cavity to fill the defect. Control of the initial corrosion rate due to the formation of a composite HYA/CMC layer on PEO can reduce the osteoblast proliferation and cellular stress; this contributes to the bone formation around the implant (Figure 8).

**Chitosan.** Chitosan (CS) is an amino polysaccharide consisting of *β*-(1→4)-linked D-glucosamine residues, and of N-acetyl-D-glucosamine (Figure 7). It is typically isolated from chitin in crustacean shells but can also be isolated from chitin of other origins (chitin from fungi, mollusks, insects, and others). Chitosan has a number of commercial and biomedical applications. It is employed in agriculture as a seed treatment and as a biopesticide to assist plants in combating fungal infections. In the field of medicine, chitosan is utilized in bandages to reduce bleeding and as an antibacterial agent; it can also be employed for transdermal drug delivery. Chitosan is distinguished by its mucoadhesive properties, which refer to its capacity to adhere to mucous membranes. Due to its cationic nature, chitosan is capable of forming insoluble polyelectrolyte complexes with anionic polymers [53,60].

Antibacterial self-compacting layer-by-layer assembled (LBL technology) chitosan-gelatin-based coatings with TiO_2_ and sodium heparin (HEP) inclusions were prepared on PEO-modified Mg-3Zn-0.5Sr alloy [38]. The three-layer films effectively sealed the pores in the PEO coating, resulting in the optimal corrosion resistance, homogeneous morphology, and robust adhesion. The incorporation of heparin into the coating led to a retardation of chitosan swelling, and the negative charge of heparin protected the sample from corrosive anions, thereby enhancing the corrosion resistance of the composite coatings. The immobilized heparin layer effectively inhibited platelet adhesion and reduced the rate of hemolysis.

Therefore, application of natural polymers to functionalize PEO coatings on Mg alloys appears to be limited.

### 2.3. Other Polymers

The use of organofluorine polymers in conjunction with inorganic PEO coating has also been studied. A composite coating was formed on magnesium alloy MA8 by plasma electrolytic oxidation in electrolyte containing Na_4_SiO_4_ and NaF, followed by treatment in an aqueous 11% suspension of super-dispersed polytetrafluoroethylene (SPTFE) (Figure 4) [39]. The anti-corrosion performance of this coating was superior to that of the base PEO layer and pure magnesium alloy. The assessment of surface wettability demonstrates that the coatings containing fluoropolymer exhibit significant hydrophobic properties. In the continuation of this work, composite Ca and P containing bioactive PEO coatings were obtained to improve the control of the resorption of the MA8 alloy [40]. The impact of super-dispersed polytetrafluoroethylene on the corrosion and wear resistance of PEO coatings containing hydroxyapatite was assessed. The results of electrochemical impedance spectroscopy and potentiodynamic polarization tests indicate that the PEO treatment resulted in a 1.7-fold increase in corrosion resistance compared to the uncoated sample. The scanning vibrating electrode technique (SVET) and the scanning ion-selective electrode technique (SIET) demonstrated the formation of pitting after a 6 h exposure of the sample to minimum essential medium (MEM). At the same time, the highest local pH value on the surface of the coating remained below 9.0. This happened due to the precipitation of hydroxyapatite-like substances that occurred as a result of the interaction between MEM components like Ca^2+^, HCO_3_^−^, and HPO_4_^2−^ with an increased concentration of OH^−^ at the corroding interface of Mg. The highest coating protection was found for the MA8/PEO/SPTFE composite polymer-containing coating, with corrosion resistance increasing by four orders of magnitude. SPTFE penetrated inside the pores, forming an additional barrier and increasing the homogeneity of the coating surface. The mechanism of corrosion development was associated with partial failure of the composite layer and clogging of the PEO coating pores by deposited corrosion products. The hydrogen release test demonstrated that the corrosion rate was reduced by a factor of 7.0 and 2.8 compared to bare and PEO-treated specimens, respectively. As a result, PEO treatment reduced the electrochemical activity of the specimen and provided biological activity of the material due to the presence of hydroxyapatite in the coating composition. The additional encapsulation of the PEO coating with SPTFE by electrophoretic deposition did not completely isolate the sample, rendering it bioinert. The coating provided limited contact of the medium with the material to ensure the bioactivity of the implant and the required rate of its decomposition.

The composite polymer-containing coatings, formed using PEO followed by impregnation with 8-hydroxyquinoline (8-HQ) and treatment with SPTFE or polyvinylidene fluoride (PVDF), exhibited the most effective anti-corrosion properties due to the controlled pore sealing [41]. The formation of protective composite coatings containing polymer and 8-HQ has been demonstrated to significantly reduce the corrosion current density of magnesium alloy (with the maximum reduction being five orders of magnitude compared to the base PEO layer). The antibacterial activity of the magnesium alloy coatings has been evaluated using a model simulating the situation where infection occurs during primary surgery. In addition, a modified assay has been developed that allows testing in the presence of a biomatrix such as blood serum. The results demonstrated that the coating containing 8-hydroxyquinoline was effective in killing methicillin-resistant *S. aureus* (MRSA) strains within 24 h. The addition of 10% human serum to the culture medium did not affect the antibacterial activity of this composite coating, suggesting a similar potential in vivo effect.

A method for the formation of composite polymer-containing coatings on magnesium alloy MA8 that involves the treatment of the sample by plasma electrolytic oxidation, followed by the application of aqueous suspension of SPTFE, was developed [42]. The coatings obtained in this study exhibited a six-order-of-magnitude reduction in corrosion current density compared to the uncoated metal and a three-order-of-magnitude reduction compared to the PEO base layer. The coatings demonstrated a significant improvement in the tribological properties of the surface layer of magnesium alloy MA8. The wear of the material was reduced by a factor of 27 compared to the PEO coating. The introduction of SPTFE from an aqueous suspension into composite coatings enables the imparting of superhydrophobic properties to the surface, resulting in a high edge angle (higher than 150°) and an edge angle hysteresis of less than 10°.

Polymer coatings based on polytrimethylene carbonate (PTMC) were prepared on the surface of magnesium alloy AZ31B (AZ/PEO/DFP) modified with PEO and the addition of dopamine [43]. A four-fold increase in corrosion resistance of AZ/PEO/DFP compared to the original AZ31B alloy was demonstrated. The osteoconductive capacity study of G292 osteoblast cells incubated directly on all samples revealed cell spreading and adhesion on AZ/PEO/DFP. Furthermore, MTT assays indicated that the coating was not cytotoxic.

Therefore, application of other polymers can improve corrosion and wear resistance of the PEO-organic coatings much higher than the combination of PEO and biocompatible widely used polymers. Moreover, this type of coating can also be non-cytotoxic and anti-bacterial.

Thus, discussing temporary metallic implants, it should be noted that the development of hybrid inorganic-organic coatings based on PEO and synthetic or natural polymers effectively addresses the issue of material corrosion resistance. This is evidenced by parameters such as corrosion potential, corrosion current, and degradation rate, as presented in Table 1. The porous structure of the PEO coating is attractive to cells; therefore, when applying the organic layer, it is important to maintain the surface morphology as well as the wettability parameter (a too hydrophobic surface will prevent cell adhesion). The organic polymeric component allows control of not only the implant dissolution rate, but also drug delivery, for which the PEO-polymer combination is a promising strategy. Encapsulating the drug within the polymer largely resolves the problems of high toxicity, low cell adhesion, and viability. Among the organic materials, natural polymers can be just as effective as synthetic ones. In some cases, they become more promising due to their unique biological properties. For example, chitosan exhibits a bacteriostatic effect due to the positive charge of its macromolecule. An interesting approach involves using organic materials, including biopolymers, that interact with ions of the dissolving metal and produce insoluble, cell-safe derivatives, which further seal defects in the coating. Analysis of research in the development of biodegradable implants shows that for each metal substrate, one must address the challenge of selecting the PEO treatment method, the type of polymer (controlling degradation rate and drug release, absence of toxicity), its thickness (for instance, excessive thickness can lead to coating detachment), and the drug concentration to ensure sufficient content in the coating, etc. The degradation process of the temporary implant should be regulated in such a way that the rate of dissolution is comparable to the rate of replacement by bone tissue. Thus, for each type of material, this is likely to be a separate problem requiring an interdisciplinary approach with the development of adequate models taking into account many factors.

## 3. Permanent Metal Implants with Organic Coatings

In contemporary medical practice, a preference is given to the permanent implants made of titanium and its alloys. This is due to the fact that these materials are bioinert and have enhanced corrosion and mechanical properties [61]. Nevertheless, the most commonly used titanium is bioinert, and it is unable to stimulate osseointegration. Furthermore, in certain instances, the application of titanium is constrained by its limited strength characteristics. In the majority of these instances, alternative materials, such as stainless steels, CoCr-alloys, and two-phase titanium alloys, including Ti-6Al-4V, remain viable options. It is important to note that these materials contain a number of alloying elements (Co, Cr, Fe, Ni) that have a negative impact on living tissues. Ti-6Al-4V alloy, for instance, contains toxic vanadium V. Therefore, a topical problem is to develop a complex approach associated with simultaneous increase in the level of mechanical properties of pure titanium and the use of protective and bioactive coatings that reduce corrosion and overcome the problem of foreign body. To address this issue, the development of composite materials, particularly biocomposite coatings on titanium implants, is being pursued [62]. Biocompatible organic coatings can promote osseointegration of implants and exhibit high osteoinductive and osteoconductive potential by incorporating into the living bone system. Alternatively, they can exhibit the property of bioinertness for the subsequent safe removal of the temporary implant [63].

The enhancement of the mechanical properties of titanium is achievable not only through the incorporation of various additives, but also through the advanced techniques of severe plastic deformation (SPD), which facilitate the transformation of the material grain structure into a nanostructured state [64]. A number of studies have demonstrated that the formation of nanostructure in titanium leads to an increase in strength and fatigue life; this helps to substitute Ti-6Al-4V alloy by a nanostructured Ti Grade 4 to manufacture modern titanium implants [65,66].

Therefore, titanium and its alloys are the focus materials for the permanent implants, so their surface treatment through plasma electrolytic oxidation and modification with organic top coating refer to a vast number of applications that require further developments of more biocompatible and bioactive devices that would be analyzed below (Table 2).

### 3.1. Synthetic Polymers

**Polyesters.** Synthetic polyesters are employed on the developed surface of Ti implants to enhance the corrosion resistance, by filling the micropores and microcracks of the surface layer. They can also act as a polymer substrate that can encapsulate a variety of compounds, including drugs, thereby enabling the controlled release of compounds and necessary biological properties [86].

To achieve the wear resistance of coatings, their adhesion to the substrate and tensile strength, as well as the controllable rate of their biodegradation and electrochemical parameters, 3D coatings made of porous calcium phosphate (CaP)-PEO on titanium substrates modified with poly (lactic-co-glycolic acid) (PLGA) have been proposed [67]. CaP coatings on Ti were deposited by ultrasonic-assisted microarc oxidation followed by PLGA modification by dip coating at concentrations of 5 wt%, 8 wt%, and 10 wt%. The addition of PLGA significantly enhanced the adhesion and cohesive strength, as demonstrated by the scratch test, while the adhesion of PLGA to CaP was at least 8.1 ± 2.2 MPa, as determined by the peel test. Tensile tests demonstrated the typical failure modes of CaP coatings, characterized by brittle fracture. The corrosion resistance of PLGA-coated CaP/Ti samples was evaluated using gravimetric and electrochemical methods in 0.9% NaCl and PBS solutions. The results indicated a significant reduction in the corrosion rate of PLGA, with the corrosion current decreased by two orders of magnitude even for the 5 wt.% PLGA/CaP/Ti sample. Furthermore, the PLGA layer demonstrated a significant increase in impedance modulus by two orders of magnitude, indicating a robust barrier against corrosion at all PLGA concentrations. Higher PLGA concentrations were found to provide even greater corrosion resistance and improved mechanical properties.

As an example, a hybrid oxide-polymer coating with an antibacterial function on the implant made of β-phase titanium alloy was obtained through the deposition of biodegradable PLGA containing the antibiotic gentamicin on the surface of Ti-15Mo alloy modified with PEO in suspension with bioactive wollastonite (CaSiO_3_) [68]. A poly (d,l-layer of lactide-co-glycolide (50/50) was formed on the PEO coated Ti-15Mo alloy surface and uniformly covered the porous oxide layer and filled the pores. The anti-corrosion properties of the coating were studied in Ringer’s solution; the study demonstrated that the polymer layer degraded uniformly, and that surface modification improved the corrosion resistance of the substrate. The oxide and polymer-oxide layers were found to be cytocompatible, with MG-63 osteoblast-like cells demonstrating attachment to the modified surfaces. Hybrid coatings comprising gentamicin and PLGA formed on the titanium alloy surface exhibited antimicrobial activity against *S. aureus*.

These studies were subsequently continued, resulting in the development of a hybrid coating based on anodized titanium alloy—Ti-15Mo, PLGA, and amoxicillin (AMX) [69]. The organic layer was deposited in such a way as to preserve the specific morphology of PEO. It was shown that the degradation process of the deposited PLGA layer took 4 weeks. The minimum inhibitory concentration of AMX was achieved for both bacterial strains *S. aureus* and *S. epidermidis* after 1 h of degradation of the PLGA/AMX coating in artificial saliva. Amoxicillin slightly decreased the MG-63 cell viability comparing to the cells on PEO and PEO/PLGA coating without the drug.

Furthermore, to investigate the advantages of the obtained polymer coating, the authors of [70] developed a hybrid oxide-polymer coatings containing a biologically active substance (doxycycline) on the surface of titanium alloy Ti-15Mo. The surface of the Ti alloy was modified using a plasma electrolytic oxidation method, followed by the formation of a polymer layer of PLGA copolymer with doxycycline (DOX). This was obtained by immersion in a solution consisting of PLGA and 10 wt.% doxycycline. The polymer uniformly coated the porous oxide layer and filled the pores. The PLGA layer was shown to hydrolyze to glycolic acid much faster than the polylactide portion to lactic acid. The adhesion and proliferation of osteoblast-like cells were observed on the modified Ti-15Mo alloy surfaces, indicating that the surface was cytocompatible. Studies using *S. aureus* (ATCC 25923) and *S. epidermidis* (ATCC 700256) strains confirmed that the amount of doxycycline loaded was sufficient to inhibit bacterial growth.

To enhance the biocompatibility of the Ti-15Mo titanium alloy, the surface of the material was modified with PEO, after which a poly(sebacic anhydride) (PSBA) (Figure 4) layer was deposited on the modified oxide layers. This layer was loaded with antibiotics amoxicillin (AMX), cefazolin (CEF), or vancomycin (VANC) [71]. The concentration of released drug was evaluated using high-performance liquid chromatography (HPLC). The results demonstrated that the amount of antibiotics administered depended on their chemical structure, molar mass, and affinity for PSBA. The HPLC results demonstrated not only the gradual release of the drug, but also the degradation of the PSBA polymer coating (51.3 and 77.8 mol% after 3 and 7 days, correspondingly). The antimicrobial efficacy of the hybrid layers against *S. aureus* (DSM 24167) and *S. epidermidis* (ATCC 700296) was demonstrated. The hybrid layer with vancomycin exhibited the greatest inhibitory effect on bacterial adhesion, while the coatings with amoxicillin and cefazolin demonstrated superior bactericidal activity.

On Ti-2Ta-3Zr-36Nb alloy, PEO-organic coatings have been developed using a polymer layer of poly(adipic anhydride) (PADA) (Figure 4) loaded with drugs such as amoxicillin, cefazolin, or vancomycin [72]. A hybrid surface layer consisting of an antimicrobial agent and a polymer on an oxidized alloy surface was prepared by immersion into a solution of 1% (wt./vol.) PADA in CHCl_3_ with the addition of one of three different antibiotics (amoxicillin, or cefazolin, or vancomycin) in an amount of 5 wt.% by weight of PADA. The degradation time of the resulting hybrid layer was measured within 48 h, and poly(adipic anhydride) was found to be a relatively rapidly degradable polymer, achieving a hydrolysis progress of more than 80% after 48 h of degradation. All coatings were shown to be cytocompatible with MG-63 osteoblast-like cells. The concentrations of drug released from the coatings were sufficient to inhibit adhesion of reference and clinical strains of *S. aureus* ATCC 25,923 and *S. aureus* MRSA 1030 bacteria. The coatings with amoxicillin showed the best results in the bacterial suppression zone, whereas the coatings with cefazolin inhibited the adhesion of the above bacteria on the surface.

**Polyethylene glycols.** One of the most well-known antifouling polymers is polyethylene glycol (PEG) (Figure 4). The majority of studies into non-fouling coatings have concentrated on the use of PEG to form branched layers on the surface [87]. This process has two main effects: firstly, steric hindrance prevents direct contact between proteins and the surface, and secondly, hydrate shells are formed around the substrate, preventing the accidental adsorption and denaturation of proteins and, therefore, the foreign body reaction. Plasma deposition of tri- and tetraethylene glycol molecules is the most common method for obtaining PEG-like surfaces [88,89]. In addition to plasma deposition, a common way to modify surfaces using PEG is covalent bonding—the step-by-step attachment of PEG containing different reactive groups. In this process, each surface requires different procedures to introduce the appropriate reactive functional groups onto the substrate so that they become susceptible to PEG [90,91,92]. The PEG layer can serve as a substrate for drugs, to respond to pH stimuli during inflammation.

PEO/PEG coatings on titanium Ti-6Al-4V loaded with betamethasone (BET), a widely used anti-inflammatory drug, were prepared to implement an approach to achieve prolonged and sustained drug release while providing reliable corrosion resistance under inflammatory conditions [73]. Application of a PEG-BET layer on TiO_2_ coatings by vacuum immersion resulted in prolonged release of pH-sensitive BET for 30 days. Electrochemical corrosion tests were conducted in both conventional and acidic inflammatory solutions to assess the protective efficacy of the duplex composite coatings. The results demonstrated that the coatings provided a 28-fold increase in protection compared to pure titanium. These findings highlight the effectiveness of the PEO/PEG-BET layer in sealing the pores within PEO coatings, thereby reducing the penetration of corrosive ions into the inflammatory environment.

A 3D-printed porous Ti-6Al-4V implant with microarc-oxidized surface loaded with vancomycin-encapsulated PEG hydrogel was developed [74]. The surface of the implant modified by micro-arc oxidation exhibited improved osteogenic activity and was well-integrated with the PEG-hydrogel drug delivery system, allowing for sustained release of vancomycin. In vitro studies highlighted excellent biocompatibility, antibacterial, and osteoinductive properties. The effective antibacterial and osteogenic capabilities of the implant were demonstrated in vivo on infected bone defects in rabbits.

Therefore, application of synthetic polymers provides reliable methods to increase the PEO-coated titanium implant corrosion resistance, provide antibacterial and anti-fouling properties to the surface, and supply the means of controlled antibiotics release.

### 3.2. Natural Polymers for Permanent Implants

**Chitosan.** To develop composite multilayer coatings with enhanced anti-corrosion and bioactive properties, the titanium surface was modified using PEO, in situ hydrothermal crystallization of hydroxyapatite and chitosan (Chi) [75]. Chitosan was selected on the basis that the reactive amine and hydroxyl groups of the polysaccharide can be utilized to impart superior corrosion resistance with self-healing properties and good adhesion. The surface treatment was conducted using the PEO method, employing an electrolyte solution comprising calcium acetate and calcium glycerophosphate. The obtained samples were subjected to hydrothermal treatment (HT) in deionized water in an autoclave (PEO/HT). The surface of PEO-modified Ti and hydrothermally treated PEO/HT was coated with chitosan from a 1% solution of the polysaccharide in acetic acid (PEO/Chi and PEO/HT/Chi). The results demonstrated that the chitosan coating over PEO and PEO/HT effectively sealed the PEO coating pores. The combined PEO/HT/Chi surface is observed to enhance corrosion resistance by a factor of two, as evidenced by *E_corr_* and *i_corr_* measurements.

One-step microarc oxidation was employed to prepare PEO-hydroxyapatite/TiO_2_ coatings on titanium. The coatings were then modified with bone morphogenic protein-2 (BMP-2) encapsulated in chitosan (BMP-2/Chi/HA/TiO_2_) [76]. The degradation ability of chitosan and the bone morphogenic protein-2 release ability, biological activity, biocompatibility, and antibacterial properties of the BMP-2/Chi/HA/TiO_2_ composite coating were investigated by in vitro tests. The results demonstrated that after applying BMP-2/Chi to the HA/TiO_2_ surface, the surface roughness and wettability decreased, while the bond strength between the HA coating and the titanium substrate improved. Chi was found to be an appropriate carrier for the delayed release of BMP-2, with the ability to continuously control the release of BMP-2 for a period of 4 weeks in a simulated human serum environment. Furthermore, Chi imparted antibacterial activity to the BMP-2/Chi/HA/TiO_2_ composite coating. The antibacterial mechanism of Chi is attributed to the positive charge on its surface, which enables it to adsorb negatively charged *E. coli*. This results in the formation of a dense polymer membrane on the cell surface, preventing the transport of nutrients into cells and the excretion of physiological metabolic wastes. This, in turn, causes metabolic disturbances and affects the growth and multiplication of bacteria. In addition to the contact mode, it has been demonstrated that Chi can also play an antibacterial role by decomposing antibacterial ions, specifically -NH_3_^+^, which were obtained by dissolution in an acidic solution. As a result, it was shown that the percent reduction of *E. coli* on Chi/HA/TiO_2_ was approximately 70% after 24 h incubation, with an antibacterial rate of approximately 30% per unit area. BMP-2/Chi/HA/TiO_2_ demonstrated the ability of cell adhesion, spreading, and proliferation. The results of cell counting (CCK-8) and SEM images showed that MC3T3-E1 cells adhered well to all surfaces, and BMP-2 administration significantly promoted the proliferation and adhesion of MC3T3-E1 cells on the surface. The loading of Chi does not appear to have a deleterious effect on the initial surface morphology and biological properties of HA/TiO_2_. This may be due to the fact that the BMP-2/Chi/HA/TiO_2_ surface has the requisite wettability, suitable surface roughness, and a large open area of HA, which are important factors that promote cell growth and proliferation on the surface.

To obtain antibacterial surface properties, hydroxyapatite and hydroxyapatite-chitosan (HA/Chi)-based coatings were prepared on the surface of PEO-modified Ti using the sol-gel method [77]. Antimicrobial studies using *E. coli* bacteria demonstrated that a dense bacterial layer was formed on and around the HA sample, whereas the density decreased on the HA/Chi sample. However, no halo of inhibition was observed around the HA/Chi sample. Consequently, the HA/Chi surfaces obtained by PEO and combined sol-gel methods were found to exhibit enhanced antibacterial properties.

In Ref. [78], molybdenum diselenide (MoSe_2_) was synthesized hydrothermally on the surface of porous TiO_2_ coating obtained by micro-arc oxidation of titanium implants to impart in situ antibacterial activity to the coating upon near infrared light (NIR) irradiation. Chitosan was adsorbed on the surface of MoSe_2_ nanosheets by electrostatic binding to improve biocompatibility. In vitro and in vivo studies demonstrated antimicrobial activity, with an efficacy of 82.03% against *Streptococcus mutans* (*S. mutans*).

**Figure 9:**
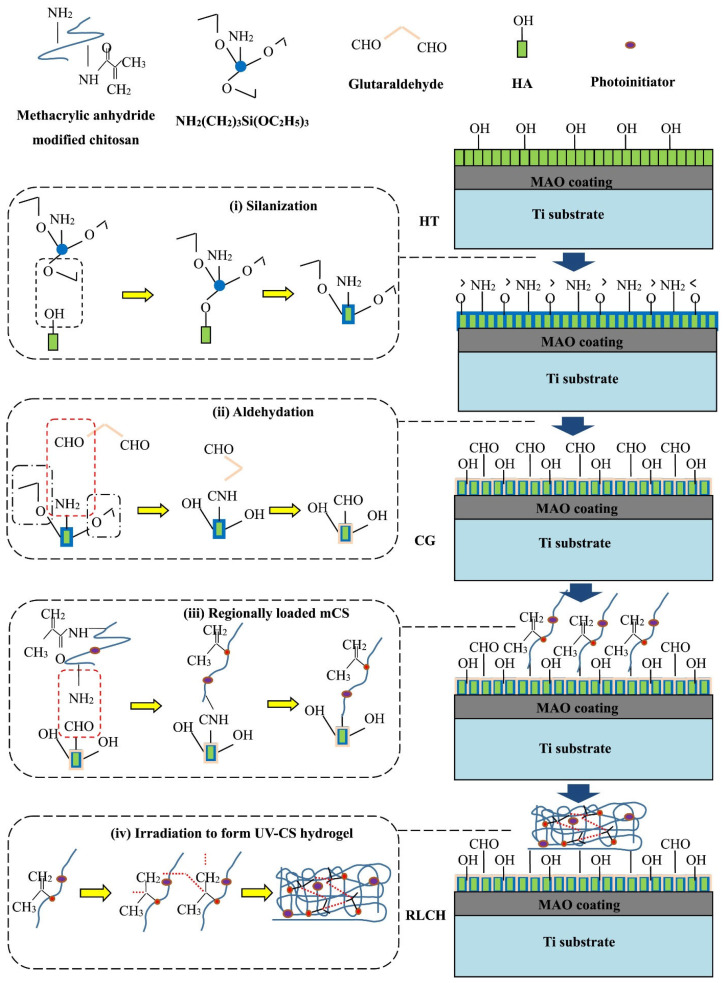
Schematic diagram of the chemical bonding between the UV-CS hydrogel and modified coating surface via chemical grafting [79].

To enhance the biological and antibacterial activity of titanium, a chitosan hydrogel (CG) containing ciprofloxacin was applied to a hydrothermally treated PEO (MAO) coating, which was coated with arrays of hydroxyapatite nanodots [79]. The PEO treatment of the Ti surface was conducted in an electrolyte containing EDTA-2Na, calcium dihydrophosphate, and NaOH. Hydrothermal treatment in NaOH solution was employed to form hydroxyapatite nanodots on the surface of the PEO-modified Ti. To enhance adhesion, surface silanization was conducted using 3-aminopropyltriethoxysilane, which was subsequently modified with glutaric aldehyde. A photocrosslinking group was grafted to the chitosan by adding methacrylic anhydride, as illustrated in Figure 9. A polydimethylsiloxane (PDMS) matrix was utilized to generate a regionally loaded chitosan hydrogel containing ciprofloxacin. Samples with different surface characteristics were immersed in simulated body fluid (SBF) to test their ability to induce hydroxyapatite formation. It was shown that by controlling the functional groups of the surface during the chemical grafting process, the modified surface not only exhibited superior ability to induce apatite formation, but also improved the adhesion between the chitosan hydrogel and PEO surface. Bacterial cultures of *E. coli* and *S. aureus* were used to study the antibacterial ability of the samples. It was found that ciprofloxacin-loaded chitosan coating on PEO-modified Ti effectively inhibited bacterial growth. The cell viability on the modified surface was tested in cell experiments using human bone marrow mesenchymal stem cells (hBMSC). The regional structure of chitosan coating with ciprofloxacin can promote cell adhesion and proliferation with superior biological activity.

**Figure 10:**
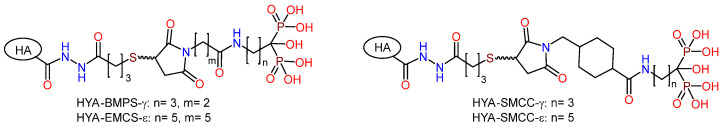
Biphosphonic derivatives of hyaluronic acid.

**Hyaluronic acid.** To obtain new biocompatible coatings on nanostructured (nano-Ti) and coarse-grained titanium (CG-Ti), the PEO sublayer was modified with bisphosphonic derivatives of natural hyaluronic acid (HYA-PH) [80]. The incorporation of phosphonate groups into the biopolymer structure was driven by the necessity to enhance the affinity of organic molecules for the oxidized metal surface and the adsorption of calcium ions. Hyaluronic acid derivatives (HYA-PH) were synthesized through the reaction of maleimide bisphosphonates of γ-aminobutanoic and ε-aminocaproic acids with SH-functionalized HYA (Figure 10). The formation of a combined coating on the surface of PEO-modified titanium was achieved through the application of organic layers due to physicochemical adsorption from aqueous solutions. The study of the biological activity demonstrated that the compounds were nontoxic; their introduction into the PEO coating resulted in a reduction in the viability of fibroblasts (by 20–40%), osteoblast-like cells (by 30–60%), and MSC (by more than 60%) on the surface. This effect appears to be due to the known ability of polysaccharides, particularly glycosaminoglycans, to form a hydrophilic hydrating layer on the surface, which prevents proteins from adsorbing on the surface, thus conferring antifouling properties [4,93]. Furthermore, a significant decrease in the adhesion of *P. aeruginosa*, *S. aureus*, and *E. faecium* bacteria on the surface of PEO-modified titanium containing HYA derivatives was observed. It has been shown that the use of nanostructured titanium leads to an increase in the content of small pores on the PEO-modified surface, which, combined with an organic coating, results in a reduction of pathogen adhesion by up to 84%.

**Peptides.** Controlling the conformation and orientation of grafted and adsorbed proteins of large sizes, as well as their production and purification, is a very complex object for study [94]. Therefore, the use of relatively short bioactive peptide sequences appears to be more promising. To improve osteointegration, various peptides of different compositions can be used; among them, biologically active coatings based on integrin-active RGD (arginine-glycine-aspartic acid) peptide occupy leading positions. RGD is an integral part of many proteins of the ECM to which cells attach using specific receptors on their surface—integrins [95,96,97]. The introduction of phosphonate groups can provide high adhesion of such peptide molecules to the implant surface, due to their high affinity for metal ions and ability to bind to the oxidized metal surface [98,99,100,101,102].

**Figure 11:**
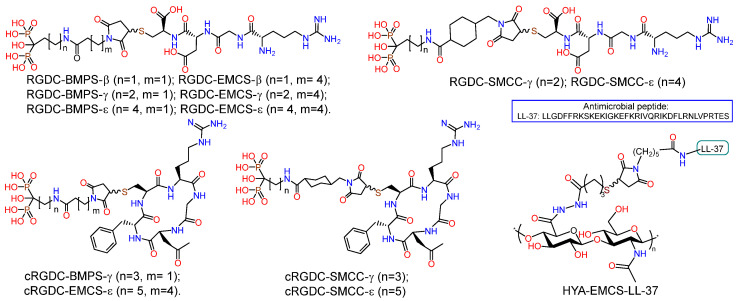
Structure of hybrid molecules based on RGD aminobisphosphonates and conjugate of HYA with LL-37.

PEO coating and RGD pore fillers on nanostructured and coarse-grained titanium substrates were prepared (Figure 11) [81]. The PEO surface treatment was conducted in an electrolyte containing Na_3_PO_4_ and Ca(CH_3_COO)_2_. Bisphosphonate derivatives (RGD-PH) of integrin-active RGDC peptide (Arg-Gly-Asp-Cys) were deposited on the PEO surface of titanium by solution immersion. It was demonstrated that the incorporation of RGD-PH into the PEO coating resulted in a reduction in surface passivation, with a subsequent decrease in the *E_corr_* value compared to the control samples. Additionally, the *i_corr_* corrosion current exhibited a significant increase. The biological activity of the PEO coating on nano-Ti was evaluated using human embryonic lung fibroblast cells (FLECH-104). The results demonstrated that the PEO coating on nano-Ti provided a 43% increase in cell number compared to CG-Ti-PEO. Furthermore, the combination of PEO coating and RGD peptides on the nano-Ti substrate provided a 45% increase in cell number compared to nano-Ti and a 66% increase in cell number compared to both uncoated and coated CG-Ti.

A series of RGD-functionalized hybrid molecules with bisphosphonate anchors and linkers (BMP, EMCS, SMCC) were prepared for the purpose of studying the structure-activity effect on the biological activity of composite coatings based on inorganic porous PEO sublayers on titanium [82]. Electrochemical studies demonstrated that following functionalization with RGD derivatives, the PEO surface exhibited a slight increase in nobility, accompanied by a notable rise in corrosion currents. Furthermore, the anodic curves of PDP on the PEO surface exhibited a loss of the passivation region following the introduction of RGD derivatives. In vitro studies on the proliferation and viability of fibroblasts, mesenchymal stem cells, and osteoblast-like cells revealed a correlation between the bioactivity of the molecule and the anchor and linker structure. In particular, RGD derivatives with relatively short bisphosphonate anchors and a BMPS linker, as well as molecules containing a linker with a cyclohexyl moiety, demonstrated enhanced cell proliferation on the PEO-modified titanium surface. RGDC, which does not contain phosphonate groups, did not affect cell proliferation on the Ti-PEO surface.

In continuation of these works, PEO coatings modified with inorganic Ca-, P-, and organic RGD aminobisphosphonates were compared in terms of their mechanical, physicochemical, electrochemical, and biological properties [83]. It has been demonstrated that a PEO-Ti coating, predominantly comprising anatase, can be formed on the Ti surface through the use of a pulsed bipolar PEO process in a sodium phosphate electrolyte. A Ca-, P- containing Ti-PEO coating can be generated in the same PEO regime in an electrolyte that contains calcium acetate; this coating contains not only titanium but also hydroxyapatite and perovskite. The Ti-PEO/RGD coating demonstrated a 37% increase in MG-63 cell proliferation compared to Ti-PEO, whereas the Ca-, P- coating of Ti-PEO exhibited a 15% decrease in proliferation.

Taking into account the selectivity of cyclic forms of RGD peptide towards integrins and their high enzymatic stability, derivatives of the cyclic peptide c(RGDfC) (cyclo(-Arg-Gly-Asp-D-Phe-Cys)) were synthesized (cRGD-PH); in the molecules, the peptide fragment was conjugated with bisphosphonates of γ-aminobutyric and ε-aminocaproic acids through BMPS, EMSC, and SMCC linkers (Figure 11) [84]. Studies of the proliferation and viability of fibroblasts, mesenchymal stem cells, and osteoblast-like cells in vitro have demonstrated that the biological activity of molecules is dependent on their structure. c(RGDfC) has been shown to reduce cell surface viability, probably due to toxicity. The introduction of the linker and bisphosphonate anchor into the structure of the molecule resulted in a reduction in the toxicity of cyclo-RGD. However, only the ε-aminocaproic acid derivative with the SMCC linker was able to enhance the degree of proliferation of fibroblasts and mesenchymal stem cells on the surface of coarse-grained or nanostructured titanium.

With the aim to reduce the risk of infectious inflammatory diseases of bacterial nature, a non-fouling antimicrobial coating HA–LL37 was developed, in which the EMCS derivative of the antimicrobial oligopeptide LL-37 was conjugated with SH-modified hyaluronic acid (Figure 11) [85]. The interest in LL-37 was due to the fact that this peptide, containing 14 amino acid residues, is a representative of the group of cathelicidins found in humans and exhibits a range of antibacterial, antiviral, and immunomodulatory activities [103,104,105]. The HYA-LL-37 conjugate was tested as an organic coating for PEO-modified coarse-grained and nanostructured titanium. In vitro studies demonstrated the antibacterial effect of the hybrid molecule as part of the inorganic PEO coating, namely a significant (*p* < 0.05) suppression of the ability of *S. aureus*, *P. aeruginosa*, *E. faecium* and *E. coli* to form biofilms.

Therefore, the application of natural polymers as pore fillers for PEO coatings on Ti implant materials opens wide possibilities to produce non-fouling and/or integrin-active coatings that contribute to the development of bioactive implantable devices of a new generation.

Consideration of methods for surface modification of permanent implants shows that PEO modification by organic molecules have a lesser impact on corrosion parameters. In this case, the hybrid coatings mainly serve functions related to drug delivery (typically, antibiotics) and ensuring necessary signaling between the device and the body to reduce inflammatory reactions and initiate the osseointegration process. This approach would be particularly valuable for treating patients with diseases that are accompanied by reduced bone mass. Thus, the formulation of further strategies must be closely linked to the establishment of biochemical mechanisms of osseointegration and the identification of relevant biological targets, which could lead to the design of new molecules that effectively manage cell adhesion, proliferation, differentiation, etc.

## 4. Conclusions and Outlook

The necessity for dental and orthopedic implants has reached a considerable scale. The most significant advancements in implant technology include enhanced strength, corrosion resistance, bioactivity, and antibacterial properties, which should facilitate accelerated osseointegration and expedite patient rehabilitation. Consequently, the most advanced scientific research in the field of biomaterials currently encompasses the development of novel implant designs based on the biomimetic approach, which aims to emulate the characteristics of living bone at all levels, including mechanical, physical, chemical, and biological.

Materials used for bone implantation must exhibit a specific interaction with the internal environment of the body and possess certain mechanical, physicochemical, and biological properties. To address this issue, composite materials are being developed, with particular focus on biocomposite coatings. Inorganic coatings formed using physicochemical methods typically exhibit weak bioactivity. Coatings obtained by organic synthesis methods provide good osteointegration, prevent the formation of fibrous tissue, and can have an antibacterial effect. However, they demonstrate poor adhesion to the substrate. Linking these two approaches is highly relevant, as it allows combining their strengths to obtain coatings with high biocompatibility, bioactivity, and mechanical performance.

Inorganic PEO coatings on metals and their alloys are inexpensive, exhibit excellent adhesion to the substrate, porosity and composition of the surface oxide layer, facilitate a gradual transition in elastic modulus from the metal implant to the bone, provide the necessary morphology for cell fixation, and serve as an excellent basis for the application of organic molecules from solutions, often without the need for additional chemical functionalization of the surface.

The design strategy for organic coatings to control biological properties of implants has been realized for many years. The intensity of interdisciplinary research in this direction is increasing, as evidenced by the growing number of publications. Organic coatings based on various classes of molecules can significantly influence cell adhesion, proliferation, and differentiation, as well as control the immune response of the organism. There is a gradual evolution of organic materials along the path of complication of composition and structure of molecules in order to realize a whole range of functions.

Future strategies for the development of temporary biodegradable devices should include the design of a coating that provides controlled degradation of the implant, as well as the release of drugs, primarily ensuring low toxicity of the substrate and coating components. In this case, a combination of PEO-inorganic coating and biopolymers with useful biological properties, including high biocompatibility, antimicrobial activity, and the ability to produce products during degradation that eliminate coating defects for a long time, is of great interest.

In turn, forthcoming design of permanent implants can be based on a personalized approach that takes into account not only the individual shape of the device, but also genomics, proteomics, and other profiles that give the information on the specific biomarkers. This would provide more precise tools for selecting appropriate agents for implant modification. In this case, the design of protein molecules, including shorter peptides, as an organic component of the implant surface would be a highly effective way to overcome the foreign body problem.

Consequently, the studies of fundamental phenomena that provide the development of biomimetic systems based on inorganic porous coatings and organic molecules that perform the function of corrosion control, provide the necessary signaling with the cells of living tissues, and possess antibacterial properties are extremely promising and can further serve as a basis for the development of new generations of implants with a wide range of purposes.

## Figures and Tables

**Table 1:** Temporary implants based on biodegradable metals.

Substrate	PEO Coating (Electrolyte Composition and Processing Mode)	Physicochemical Properties of PEO Coating (Porosity, Corr. Resistance, Thickness, etc.)	Organic Coating	Physicochemical Properties of Hybrid Coating PEO + Organic Molecules	Biological Effect of Hybrid Coating	Refs.
Mg-1.21Li-1.12Ca-1.0Y	NaOH, Na_2_SiO_3_, NaB_4_O_7_, Na_3_C_6_H_5_O_7_, phytic acid; constant voltage 130–140 V, t = 10 min, room temperature	*d_mean_* = 8.61 μm, number of pores (NP): 15,332, corrosion current density *i_corr_* = 6.313 × 10^−6^ A·cm^−2^, charge transfer resistance *R*_ct_ = 354.8 Ω/cm^2^	PLA (Mw = 60,000)	*d_mean_* = 2.26 μm, NP: 3489, *i_corr_* = 1.702 × 10^−6^ A·cm^−2^, *R*_ct_ = 1894.0 Ω/cm^2^	-	[27]
AZ31	Na_2_SiO_3_·9H_2_O, NaOH, NaF; constant current density 50 mA·cm^−2^, frequency 800 Hz, duty cycle 20%, t = 15 min, room temperature	*E_corr_* = −1.606 V, *i_corr_* = 1.1 μA·cm^−2^, *R*_c_ = 4888 Ω/cm^2^, *R*_ct_ = 58,295 Ω/cm^2^	PLA	*E_corr_* = −1.317 V, *i_corr_* = 0.00024 μA·cm^−2^ *R*_c_ = 32,892 Ω/cm^2^, *R*_ct_ = 895,900 Ω/cm^2^	Hemolysis ratio (%): 0.806 ± 0.771 (<5%, excellent blood compatibility). Adhesion (1 day): The MC3T3-E1 cells exhibited good adhesion and displayed numerous filopodia extensions. Proliferation and vitality (3 and 7 days): MC3T3-E1 cells on PEO/PLA coating showed higher proliferation rate and vitality than that on PEO coating.	[28]
Mg-1Li-1Ca	NaOH, phytic acid; breakdown voltage 170–180 V, duty cycle 50%, frequency 100 Hz, t = 600 s	*E_corr_* = −1.62 V, *i_corr_* = 1.03 × 10^−5^ A·cm^−2^ (sample immersion 140 h)	PLA (Mw = 200,000)	*E_corr_* = −1.60 V, *i_corr_* = 4.19 × 10^−6^ A·cm^−2^	Hemolysis ratio (%): 0.17 ± 0.04 (<5%, excellent blood compatibility). Cytotoxicity: MC3T3-E1 cells, MTT assay, relative growth ratio (RGR) of all samples is <100% after 24 h of culture, RGR of MAO/PLA coating >100% after 72 h. Proliferation: MAO/PLA showed higher proliferation rate and vitality comparing with Mg-1Li-1Ca substrates and MAO coating. Alkaline Phosphatase (ALP) activity (osteoblastic differentiation): MAO/PLLA coatings displayed a highest ALP expression of MC3T3 cells	[29]
AZ31	Na_3_PO_4_·12H_2_O, NaF, KOH, CaO; peak-to-peak voltage 480 V and direct current offset 190 V, duty cycle 50%, frequency 50 Hz, current density limit 138 mA·cm^−2^	average bonding strength 5.2 ± 0.4 MPa, *E_corr_* = −1.23 V, *i_corr_* = 6.5 × 10^−8^ A·cm^−2^	PLA	average bonding strength 4.0 ± 0.7 MPa, *E_corr_* = −1.13 V, *i_corr_* = 1.7 × 10^−8^ A·cm^−2^	-	[30]
Mg	Na_2_SiO_3_·9H_2_O, NaF; current density limit 50 mA/cm^2^, limiting voltage 250 V, t = 3 min	*E_corr_* = −1.65 V, *i_corr_* = 1.14 μA·cm^−2^	PCL (Mw = 70,000–90,000)	*E_corr_* = −1.53 V, *i_corr_* = 0.0045 μA·cm^−2^	-	[31]
Mg, screw	NaOH, Na_3_PO_4_, glycerol; constant current 300 mA/cm^2^, pulse width 100 ms, duty cycle 50%, t = 3 min	-	PCL (Mw = 70,000–90,000)	-	In vivo: Sprague Dawley rats, stable and compact new bone formed on the PCL coating. The hybrid coating reduced the release of magnesium ions and degradation rate of magnesium. Denser and thicker bone formed around the PEO/PCL-coated screw than the PEO-coated screw after 2 months of implantation.	[32]
AZ31	Na_2_SiO_3_·9H_2_O, KOH, KF·2H_2_O; current density 50 mA/cm^2^, frequency 300 Hz, duty cycle 10%, t = 15 min	*E_corr_* = −1.640 V, *i_corr_* = 4.738 × 10^−7^ A·cm^−2^, *R_p_* = 1.930 × 10^5^ Ω	PCL, PCL/PDAM, PCL/PDAM/ PHMB	PCL: *E_corr_* = −1342 V, *i_corr_* = 3.260 × 10^−10^ A·cm^−2^, *R_p_* = 2.686 × 10^8^ Ω; PCL/PDAM: *E_corr_* = −1.303 V, *i_corr_* = 1.843 × 10^−10^ A·cm^−2^, *R_p_* = 4.920 × 10^8^ Ω	Cytotoxicity: MC3T3-E1 cells, viability >70%. Adhesion (1 day): cells were fully spread and displayed polygon shapes with a large number of filopodia and lamellipodia on PEO/PCL and PEO/PCL/PDAM surfaces compared to AZ31 and AZ31-PEO. Proliferation (7 days): cells on PEO/PCL and PEO/PCL/PDAM formed a layer, the amounts of cells on the AZ31 alloy and PEO were small. Antibacterial efficacy: *S. aureus*, *E. coli*, PEO/PCL and PEO/PCL/PDAM did not show antibacterial effect. The effect was achieved after PHMB immobilization on the surface.	[33]
Mg0.8Ca	Na_3_PO_4_·12H_2_O, Na_2_SiO_3_·5H_2_O, KOH, CaO, NaF; peak-to-peak voltage 490 V, direct current offset 190 V, duty cycle 50%, frequency 50 Hz, current density limit 138 mA·cm^−2^, t = 300 s	Thickness ≈ 13 µm, Roughness *R_a_* = 0.84 ± 0.01 µm, *R_z_* = 5.6 ± 0.3 µm, contact angle 34.1 ± 4.6°	PCL/BF	Thickness ≈ 35 µm, *R_a_* = 8 ± 0.30 µm, *R_z_* = 36 ± 0.6 µm, contact angle 106.3 ± 1.8°	Adhesion and proliferation: C2C12-GFP (ATCC CRL-1772) mouse premyoblast cell line, cells can colonize the inner PEO ceramic coating structure where higher amount of bioelements are present. The Mg/PEO/PCL/BF scaffolds exhibit equally good or better cell adhesion and proliferation compared with Ti CP control.	[34]
Mg3Zn0.4Ca	Na_3_PO_4_·12H_2_O, Na_2_SiO_3_·5H_2_O, KOH, CaO; peak-to-peak voltage 400 V, frequency 50 Hz, current density limit 100 mA·cm^−2^, t = 300 s	-	PCL/BF, PCL/BF/CIP	PCL/BF: pore size 8.5 ± 2.8 µm, contact angle 88.9 ± 5.0° PCL/BF/CIP: pore size 12.2 ± 1.1 µm, contact angle 96.1 ± 3.0°	PCL/BF/CIP system avoided burst release and ensured gradual drug elution (64% over 240 h). Over 11 days of immersion in pseudo-physiological conditions (at constant pH 7.4 under CO_2_ flow), the PCL/BF/CIP system revealed 74% reduction of the degradation rate of Mg alloy compared to the PCL/BF.	[35]
Mg3Zn0.4Ca	Na_2_SiO_3_·5H_2_O, Na_3_PO_4_·12H_2_O, CaO, KOH; peak-to-peak voltage 400 V, frequency 50 Hz, current density limit 100 mA·cm^−2^, t = 300 s	-	PCL/BF/CIP PCL/BF/PAR		Drug release: for the loaded Mg-HHC system, a gradual elution with 20 and 40% of PAR, and CIP was observed after 10 days. Cytotoxicity: C2C12-GFP (ATCC CRL-1772) mouse premyoblast cell line and C166-GFP (ATCC CRL-2583) mouse endothelial cell line, the addition of PAR did not prevent the growth of endothelial cells and premyoblasts in the range of concentrations up to 120 μg/mL. PAR and CIP release from a complete Mg-HHC system showed a lower concentration range, suggesting that drug addition would not constitute a drawback in the biomaterial’s cytocompatibility.	[36]
ZM21	Na_2_SiO_3_·9H_2_O, KOH, Na_2_B_4_O_7_·10H_2_O; current density 60 mA/cm^2^, duty cycle 50%, frequency 1000 Hz, t = 10 min	Thickness 28 ± 2 μm, *R_a_* = 2.085 μm, contact angle 40.2 ± 1.5°, *E_corr_* = −549.1 ± 38.2 mV, *i_corr_* = (1.48 ± 0.13) × 10^−5^ mA·cm^−2^, corrosion rate (3.81 ± 0.33) × 10^−4^ mm/y	PCL (Mw = 80,000)	PCL5: Thickness 68 ± 5 μm, *R_a_* = 1.529 μm, contact angle 63.2 ± 1.3°, *E_corr_* = 543.2 ± 45.3 mV, *i_corr_* = (6.47 ± 0.52) × 10^−9^ mA·cm^−2^, corrosion rate (1.67 ± 0.14) × 10^−7^ mm/y	Cytotoxicity: mouse fibroblast cells L929. The ZM21/PEO/PCL5 material exhibited the highest biological activity with a significantly lower number of dead cells even at 100% extract concentration. The sample demonstrated superior cell growth and proliferation in the direct contact assay.	[37]
Mg	NaOH, Na_3_PO_4_, glycerol; unipolar pulse current 300 mA/cm^2^, duty cycle 50%, t = 3 min	surface roughness *Rq* = 109.1 ± 8.69 nm, contact angle 28.601 ± 0.68°, *E_corr_* = −1.55 V, *i_corr_* = 5.362 × 10^−6^ A·cm^−2^	HYA/CMC	*Rq* = 180 ± 31.01 nm, contact angle 16.192 ± 0.46°, *E_corr_* = −1.034 V, *i_corr_* = 5.362 × 10^−7^ A·cm^−2^	In vitro: Osteoblast cells (MC3T3-E1), HYA/CMC significantly increased the cell proliferation and viability in 3-day test. In vivo: Sprague Dawley rats, HYA/CMC loading of the PEO layer self-heals localized damage and stable osteocytes in long-term bone regeneration; the layer reduced osteoblast proliferation and cell stress, and this stably promotes bone formation around the implant.	[25]
Mg-3Zn-0.5Sr	Na_5_P_3_O_10_, NaOH, C_3_H_8_O_3_, NaF, K_2_TiF_6_,CH_3_COOAg	*E_corr_* = −1.681 V vs. SCE, *i_corr_* = 7.659 × 10^−4^ A·cm^−2^, polarization resistance *R_p_* = 219.6 Ω·cm^2^	nano-TiO_2_/CS/GEL/HEP	*E_corr_* = −1.505 V vs. SCE, *i_corr_* = 2.776 × 10^−7^ A·cm^−2^, *R_p_* = 130,830.6 Ω·cm^2^	Immobilized heparin layer effectively inhibited the adhesion of platelets and reduced the hemolysis rate.	[38]
MA8	Na_4_SiO_4_, NaF; 1st step: voltage 30 to 240 V, sweep rate 1.05 V/s, t = 200 s; 2nd step: voltage 240 to 200 V, sweep rate 0.07 V/s, t = 600 s, duty cycle 50%, frequency 300 Hz	contact angle 50°	SPTFE	contact angle 155°	-	[39]
MA8	C_3_H_7_O_6_PCa, NaF, Na_2_SiO_3_; pulsed bipolar mode, voltage 420 V, duty cycle 50%, frequency 100 Hz, t = 110 s	*E_corr_* = −1.57 V vs. SCE, *i_corr_* = 5.4 × 10^−6^ A·cm^−2^	SPTFE	*E_corr_* = −0.18 V vs. SCE, *i_corr_* = 7.6 × 10^−10^ A·cm^−2^	-	[40]
MA8	(1) SiF electrolyte: Na_2_SiO_3_·5H_2_O, NaF; pulsed bipolar mode, voltage 30 to 300 V (anode) and 30 V (cathode), sweep rate 0.45 V·s^−1^, t = 10 min (2) GP electrolyte: (C_3_H_7_O_6_P)Ca_2_·H_2_O, NaF, Na_2_SiO_3_·5H_2_O; pulsed bipolar mode, voltage 380 V (anode), current density 1.3 to 0.8 A·cm^−2^, sweep rate—4.5 mA·cm^−2^·s^−1^, t = 110 s	SiF electrolyte: *E_corr_* = −1.48 V vs. SCE, *i_corr_* = 1.5 × 10^−7^ A·cm^−2^, GP electrolyte: *E_corr_*= −1.62 V vs. SCE, *i_corr_* = 2.2 × 10^−7^ A·cm^−2^	8-HQ/SPTFE, 8-HQ/PVDF	SiF electrolyte: 8-HQ/SPTFE, *E_corr_* = −1.42 V vs. SCE, *i_corr_* = 7.7 × 10^−12^ A·cm^−2^; 8-HQ/PVDF, *E_corr_* = −1.22 V vs. SCE, *i_corr_* = 1.6 × 10^−11^ A·cm^−2^; GP electrolyte: 8-HQ/SPTFE, *E_corr_* = −1.24 V vs. SCE, *i_corr_* = 9.2 × 10^−12^ A·cm^−2^; 8-HQ/PVDF, *E_corr_* = −1.19 V vs. SCE, *i_corr_* = 1.5 × 10^−11^ A·cm^−2^;	Antibacterial efficacy: 8-HQ coating killed *S. aureus* (MRSA) within 24 h.	[41]
MA8	Na_4_SiO_4_, NaF; 1st step: voltage 20 to 240 V, t = 200 s; 2nd step: voltage 240 to 200 V, sweep rate 0.07 V/s, t = 600 s, duty cycle 50%, frequency 300 Hz	*E_corr_* = −1.43 V, *i_corr_* = 2.4 × 10^−7^ A·cm^−2^, contact angle 45.3 ± 1.2°	SPTFE	*E_corr_* = 0.12 V, *i_corr_* = 7.7 × 10^−11^ A·cm^−2^, contact angle 152.3 ± 0.9	-	[42]
AZ31B	KOH, Na_3_PO_4_·12H_2_O, current density 50 mA·cm^−2^, duty cycle 25%, t = 15 min	*E_corr_* = −1.81 V, *i_corr_* = 4.31 × 10^−5^ A·cm^−2^, porosity 1.06 × 10^−1^%, contact angle 65 ± 6.4°	DFP (dopamine functionalized PTMC polymer)	*E_corr_* = −1.58 V, *i_corr_* = 0.56 × 10^−7^ A·cm^−2^, porosity 2.53 × 10^−4^%, contact angle 41 ± 3.6°	Cell viability: osteoblast-like cells (G292), PEO/DFP sample showed the highest cell viability, indicating no significant toxicity in time intervals (1, 3, and 8 days). Proliferation: the highest cell spreading as well as confluence was observed in the case of PEO/DFP coating.	[43]

**Table 2:** Permanent Metal Implants with Organic Coatings.

Substrate	PEO Coating (Electrolyte Composition and Processing Mode)	Physicochemical Properties of PEO Coating (Porosity, Corr. Resistance, Thickness, etc.)	Organic Coating	Physicochemical Properties of Hybrid Coating PEO + Organic Molecules	Biological Effect of Hybrid Coating	Refs.
Ti (Grade 2)	Nanosized hydroxyapatite (Ca_10_(PO_4_)_6_(OH)_2_), CaCO_3_, H_3_PO_4_; voltage 200 V, frequency 50 Hz, pulse duration 100 μs, t = 10 min	0.9% NaCl solution: *E_corr_* = −0.065 mV, *i_corr_* = 228.2 × 10^−9^ A·cm^−2^ PBS solution: *E_corr_* = −0.088 mV, *i_corr_* = 355.0 × 10^−9^ A·cm^−2^, adhesion strength 20.1 ± 1.6 MPa, roughness *R_a_* = 3.3 ± 0.4 μm	5%, 8%, 10% PLGA	0.9% NaCl solution (8% PLGA): *E_corr_* = −0.508 mV, *i_corr_* = 0.3 × 10^−9^ A·cm^−2^; PBS solution (10% PLGA): *E_corr_* = −0.416 mV, *i_corr_* = 0.1 × 10^−9^ A·cm^−2^, adhesion strength 9.8 ± 3.8 MPa (8% PLGA), *R_a_* = 3.0 ± 0.3 μm (8% PLGA)	-	[67]
Ti-15Mo	Ca(H_2_PO_2_)_2_, CaSiO_3_; voltage 300 V, current density 100 mA·cm^−2^	*E_corr_* = 178.7 ± 8.1 mV, *i_corr_* = (6.7 ± 0.4) × 10^−6^ A·cm^−2^	PLGA, PLGA/ gentamicin	PLGA: *E_corr_* = −168.8 ± 4.3 mV, *i_corr_* = (3.5 ± 0.1) × 10^−5^ A·cm^−2^	Cell viability: MG-63, PEO/PLGA layers were cytocompatible, and cells were well-adhered to the modified surfaces. Antibacterial efficacy: *S. aureus* (DSM 24167), for the PEO/PLGA samples, slightly higher adhesion area of *S. aureus* was observed, gentamicin-loaded PLGA samples showed no adhesion of bacteria.	[68]
Ti-15Mo	Ca(H_2_PO_2_)_2_; voltage 300 V, current density 100 mA·cm^−2^, t = 5 min	*R_a_* = 1.20 μm, contact angle 44.7 ± 5.9°	PLGA, PLGA/AMX	PLGA: *R_a_* = 1.46 μm, contact angle 91.4 ± 4.3°; PLGA/AMX: *R_a_* = 1.71 μm, contact angle 96.5 ± 2.2°	Cell viability: MG-63, after 7 days of culture, the highest level of cell viability was observed for Ti-15Mo and Ti-15Mo/PEO. PLGA and PLGA/AMX layers provided a slightly lower number of cells compared to Ti-15Mo and Ti-15Mo/PEO. Antibacterial efficacy: *S. aureus* (DSM 24167), *S. epidermidis* (ATCC 700296, the concentration of released drug during 1 h was enough to inhibit growth of both bacteria strains.	[69]
Ti-15Mo	Ca(H_2_PO_2_)_2_; voltage 300 V, current density 100 mA·cm^−2^ t = 5 min	surface roughness *R_z_* = 1.5 ± 0.3 μm, *R_a_* = 0.5 ± 0.09 μm	PLGA (M_n_ 19,000), PLGA/DOX	PLGA: *R_z_* = 2.3 ± 0.9 μm, *R_a_* = 1.2 ± 0.2 μm; PLGA/DOX: *R_z_* = 4.9 ± 0.9 μm, *R_a_* = 1.2 ± 0.1 μm	Cell viability: MG-63, after 7 days, the highest increase in the cell number was observed for PEO/PLGA–21.17%. For PEO/PLGA/DOX the proliferation decreased to 10.30%, but it was higher than in the case of Ti-15Mo and Ti-15Mo/PEO. Antibacterial efficacy: *S. aureus* (ATCC 25923), *S. epidermidis* (ATCC 700256), the amount of loaded doxycycline was sufficient to inhibit bacterial growth.	[70]
Ti-15Mo	Ca(H_2_PO_2_)_2_; voltage 300 V, current density 100 mA·cm^−2^, t = 5 min	*R_a_* = 1.21 ± 0.15 μm, contact angle 47.6 ± 5.7°	PSBA, PSBA/AMX, PSBA/CEF, PSBA/VANC	PSBA: *R_a_* = 0.82 ± 0.17 μm, contact angle 80.6 ± 2.1°; PSBA/AMX: *R_a_* = 0.81 ± 0.09 μm, contact angle 62.9 ± 3.9°; PSBA/CEF: *R_a_* = 0.93 ± 0.19 μm, contact angle 74.6 ± 3.7°; PSBA/VANC: *R_a_* = 1.2 ± 0.05 μm, contact angle 79.0 ± 2.2°	PSBA undergoes rapid hydrolysis (77.8 mol% after 7 days). Antibacterial efficacy: *S. aureus* (DSM 24167) and *S. epidermidis* (ATCC 700296), all of the coatings significantly decreased the number of bacteria, and the concentration of the drugs was suitable for septic treatment around the material. The inhibition of bacteria growth depends on the concentration of drug released from the coatings. Amoxicillin showed the best results for the artificial saliva.	[71]
Ti-2Ta-3Zr-36Nb	Ca(H_2_PO_2_)_2_; voltage 300 V, current density 150 mA·cm^−2^, t = 5 min	*R_a_* = 1.24 ± 0.35 μm, contact angle 61.31°	PADA, PADA/AMX, PADA/CEF, PADA/VANC	PADA: *R_a_* = 1.29 ± 0.05 μm, contact angle 41.69°; PADA/AMX: *R_a_* = 1.53 ± 0.26 μm, contact angle 63.02°; PADA/CEF: *R_a_* = 1.92 ± 0.28 μm, contact angle 51.24°; PADA/VANC: *R_a_* = 2.06 ± 0.36 μm, contact angle 39.51°	PADA is the fast-degrading polymer (80% after 48 h). Cell viability: MG-63, the viability of cells on the hybrid layers was slightly higher than on the PEO surface. After 7 days PEO/PADA/AMX showed the highest number of cells in comparison to other drug-loaded surfaces. Antibacterial efficacy: *S. aureus* (ATCC 25923) and *S. aureus* (MRSA 1030). Surfaces loaded with drug exhibited stronger bacteriostatic effects than those without antibiotics. The minimal number of adhered bacteria was observed for PEO/PADA/CEF.	[72]
Ti-6Al-4V	K_2_SiO_3_, KOH; constant current density 75 mA·cm^−2^, duty cycle 50%, t = 60 min	test’s solution, normal (pH 7.2): *E_corr_* = −206 mV, *i_corr_* = 2.14 μA·cm^−2^; inflammatory (pH 5.0): *E_corr_* = −329 mV, *i_corr_* = 8.87 μA·cm^−2^	PEG (Mw = 1000 g·mol^−1^)/BET	normal (pH 7.2): *E_corr_* = −54 mV, *i_corr_* = 0.081 μA·cm^−2^; inflammatory (pH 5.0): *E_corr_* = −154 mV, *i_corr_* = 1.75 μA·cm^−2^	-	[73]
Ti-6Al-4V (3D printed)	Ca(CH_3_COO)_2_·H_2_O, NaH_2_PO_4_, EDTA-2Na, NaOH	-	PEG hydrogel, PEG-VANC	-	Cell adhesion and proliferation: hMSCs, no significant difference was found in proliferative activity between PEO, PEO/PEG, PEO/PEG-VANC. PEO/PEG showed higher ALP activity compared to the other two groups, VANC reduced hMSC osteo-differentiation. Antibacterial efficacy: *S. aureus*, PEO/PEG-VANC implants showed significantly greater effect comparing with PEO and PEO/PEG. In vivo: male New Zealand white rabbits, after 6 weeks intraporous bone volume fraction (BVF) of the PEO/PEG-VANC group (49.2% ± 3.3%), was significantly higher than that of the other two groups (PEO 14.6% ± 2.2%, PEO/PEG 16.4% ± 3.2%). Histological studies of infected sites in the PEO and PEO/PEG groups showed typical signs of chronic bone infection, including inflammatory cell (mononuclear cell and granulocyte) infiltrate, bone necrosis, and bone erosion. These features were notably diminished in the PEO/PEG-VANC group.	[74]
Ti	(1) PEO: Ca(CH_3_COO)_2_, calcium glycerophosphate; voltage 350 V, current density 50 mA·cm^−2^, t = 10 min; (2) hydrothermal treatment, T = 250 °C, t = 3 h in an autoclave	(1) *R_a_* = 0.328 ± 0.012 μm, *E_corr_* = −0.288 V, *i_corr_* = 2.93 × 10^−7^ A·cm^−2^; (2) *R_a_* = 0.513 ± 0.011 μm, *E_corr_* = −0.229 V, *i_corr_* = 2.33 × 10^−7^ A·cm^−2^	Chitosan (Mw = 200 kDa, ≥93% deacetylated)	(1) *R_a_* = 0.422 ± 0.016 μm, *E_corr_* = −0.194 V, *i_corr_* = 1.96 × 10^−7^ A·cm^−2^; (2) *R_a_* = 0.649 ± 0.029 μm, *E_corr_* = −0.182 V, *i_corr_* = 1.83 × 10^−8^ A·cm^−2^	-	[75]
Ti	Ca(COOH)_2_, NaH_2_PO_4_; voltage 360 V, frequency 100 Hz, t = 5 min	HA/PEO: *R_a_* = 0.774 ± 0.023 μm, contact angle 16.4 ± 1.5°	BMP-2, Chitosan, BMP-2/Chitosan	BMP-2: *R_a_* = 0.759 ± 0.016° μm, contact angle 17.1 ± 1.2; Chitosan: *R_a_* = 0.634 ± 0.017° μm, contact angle 21.7 ± 0.8; BMP-2/Chitosan *R_a_* = 0.622 ± 0.026 μm, contact angle 22.1 ± 1.0°	Chitosan completely degrades on the surface within 4 weeks in a PBS—lysozyme solution. BMP-2/HA/PEO surfaces released about 95% of the BMP-2 within 2 days. BMP-2 released from BMP-2/Chi/HA/PEO for 4 weeks in a PBS—lysozyme solution and somewhat slower in PBS solution. Cell proliferation: MC3T3-E1, BMP-2/Chi/HA/PEO showed highest cell proliferation in a 7-day test. Antibacterial efficacy: *E. coli*, Chi/HA/PEO exhibited strong antibacterial activity (70% decrease after 24 h incubation).	[76]
Ti	(1) PEO: Na_2_SiO_3_, TiO_2_; voltage 400 V, t = 5 min. (2) HA deposition: Ca(OH)_2_ + H_3_PO_4_.		Chitosan		Antibacterial efficacy: *E. coli*, more intense bacteria growth was obtained on the PEO/HA surface compared to that of PEO/HA/Chi.	[77]
Ti	(1) PEO: Ca(CH_3_COO)_2_, Na_3_PO_4_·12H_2_O, current density 20 A·dm^−2^, t = 5 min, pulse frequency 800 Hz, duty ratio 30%, 25 °C (2) MoSe_2_ deposition: Na_2_MoO_4_·2H_2_O, Se powder, hydrothermal treatment	PEO/MoS_2_: contact angle 130°	Chitosan	contact angle 40°	Cell viability: MC3T3-E1, hydrophobic PEO/MoS_2_ is not favorable to the cell adhesion and spreading. The cells regained the vitality and spread again on PEO/MoSe_2_/Chi surface. Antibacterial efficacy: *S. mutans*, PEO/MoSe_2_/Chi exhibited excellent antibacterial activity in vitro and in vivo (82.03%) upon illumination with 808 nm NIR light. The coating promoted new bone formation in the presence of infection in vivo under NIR light irradiation.	[78]
Ti	(1) PEO: EDTA-2Na, Ca(H_2_PO_4_)_2_·H_2_O, NaOH; voltage 350 V, frequency 1 kHz, duty ratio 10%, t = 5 min; (2) HA nanodots: NaOH, hydrothermal treatment	(1) *R_a_* = 0.102 ± 0.014 μm; (2) *R_a_* = 0.140 ± 0.008 μm	Surface silanization, aldehydation (CG), Chitosan (UV-CS)/ciprofloxacin	PEO/HT/CG *R_a_* = 0.133 ± 0.004 μm	Cell viability: hBMSCs, regional loading chitosan hydrogel with ciprofloxacin does not show any biological toxicity. Antibacterial test: *E. coli*, *S. aureus*, the synergistic effect of ciprofloxacin and modified chitosan allows to effectively inhibit bacterial growth with low drug loadings.	[79]
CG-Ti, nano-Ti	Na_3_PO_4_·12H_2_O; pulsed bipolar mode, positive pulse: voltage 470 V, duty cycle 51%, negative pulse: voltage 40 V, duty cycle 26%, frequency 300 Hz, 20 °C, t = 5 min	CG-Ti/PEO: *R_a_* = 2.6 ± 0.13 μm, porosity 7.6 ± 1.4%, average pore size 1.44 ± 0.17 μm, contact angle 67°; nano-Ti/PEO: *R_a_* = 2.5 ± 0.05 μm, porosity 7.6 ± 1.5%, average pore size 0.61 ± 0.11 μm, contact angle 68°	HYA, HYA-PH	CG-Ti/PEO/HYA-PH: contact angle 60°; nano-Ti/PEO/HYA-PH: contact angle 51°	Cytotoxicity: human adipose tissue MSC, HYA and all HYA-PH are non-toxic, some HYA-PH promote cell growth. Cell viability: PEO/HYA and PEO/HYA-PH provided a reduction in the viability of fibroblasts (by 20–40%), MG-63 (by 30–60%), and MSC (by more than 60%) on the surface (7-day test). Antibacterial efficacy: *P. aeruginosa*, *S. aureus*, *E. faecium*. The significant decrease in the adhesion of bacteria on PEO/HYA and PEO/HYA-PH surface was found. Use of nano-Ti as a substrate resulted in a reduction of pathogen adhesion by up to 84%.	[80]
CG-Ti, nano-Ti	Na_3_PO_4_·12H_2_O, Ca(CH_3_COO)_2_, pulsed bipolar mode, positive pulse: voltage 470 V, duty cycle 51%, negative pulse: voltage 40 V, duty cycle 26%, frequency 300 Hz, 20 °C, t = 10 min	CG-Ti/PEO: *R_a_* = 1.13 ± 0.09 μm, porosity 8.8 ± 0.5%, average pore size 3.3 ± 0.6 μm, *E_corr_* = 0.033 ± 0.029 V, *i_corr_* = (7.7 ± 1.2) × 10^−9^ A·cm^−2^; nano-Ti/PEO: *R_a_* = 0.75 ± 0.11 μm, porosity 10.2 ± 0.5%, average pore size 3.1 ± 0.6 μm, *E_corr_* = −0.169 ± 0.022 V, *i_corr_* = (35.1± 2.2) × 10^−9^ A·cm^−2^	RGD-PH	CG-Ti/PEO/RGD-PH: *E_corr_* = −0.338 ± 0.031 V, *i_corr_* = (176 ± 32.1) × 10^−9^ A·cm^−2^; nano-Ti/PEO/RGD-PH: *E_corr_* = −0.419 ± 0.026 V, *i_corr_* = (168 ± 49.1) × 10^−9^ A·cm^−2^	Cell viability: human embryonic lung fibroblasts (FLECH-104). PEO coating on nano-Ti gives 43% increase in the number of cells compared to CG-Ti/PEO; nano-Ti/PEO/RGD-PH gives 45% increase in the number of cells compared to nano-Ti, and 66% compared to both uncoated and coated CG-Ti.	[81]
Ti	Na_3_PO_4_·12H_2_O; pulsed bipolar mode, positive pulse: voltage 470 V, duty cycle 51%, negative pulse: voltage 40 V, duty cycle 26%, frequency 300 Hz, 20 °C, t = 5 min	*E_corr_* = −0.238 V, *i_corr_* = 0.019 μA·cm^−2^	RGDC, RGD-PH	*E_corr_* = −0.218 – −0.180 V, *i_corr_* = 0.179 – 0.401 μA·cm^−2^	Cell viability: FLECH-104, MSC, MG-63. RGDC does not influence cell viability on PEO surface. RGD-PH with relatively short bisphosphonate anchors and BMPS linker, as well as molecules containing a linker with a cyclohexyl fragment, increase cell viability on the surface of PEO-modified titanium.	[82]
Ti	E1: Na_3_PO_4_·12H_2_O; E2: Na_3_PO_4_·12H_2_O, Ca(CH_3_COO)_2_; pulsed bipolar mode, positive pulse: voltage 470 V, duty cycle 51%, negative pulse: voltage 40 V, duty cycle 26%, frequency 300 Hz, 20 °C, t = 5 min	E1: *R_a_* = 2.1 ± 0.4 μm, porosity 6.8 ± 0.4%, average pore size 0.82 ± 0.17 μm, *E_corr_* = 0.1024 ± 0.005 V, *i_corr_* = (9.45 ± 0.8) × 10^−9^ A·cm^−2^; E2: *R_a_* = 3.1 ± 0.5 μm, porosity 14.5 ± 0.4%, average pore size 0.77 ± 0.16 μm, *E_corr_* = 0.0331 ± 0.029 V, *i_corr_* (A·cm^−2^) = (7.67 ± 1.2) × 10^−9^ A·cm^−2^	RGD-PH	PEO(E1)/RGD-PH *E_corr_* = −0.218 ± 0.001 V, *i_corr_* = (2.16 ± 1.12) × 10^−7^ A·cm^−2^	Cell viability: MG-63, cell viability decreased by 15% over 7 days on the surface of sample PEO(E2) compared to sample PEO(E1); the bioactivity of PEO(E1)/RGD-PH is 37% higher compared to PEO(E1).	[83]
CG-Ti, nano-Ti	Na_3_PO_4_·12H_2_O, pulsed bipolar mode, positive pulse: voltage 470 V, duty cycle 51%, negative pulse: voltage 40 V, duty cycle 26%, frequency 300 Hz, 20 °C, t = 5 min	CG-Ti/PEO: *R_a_* = 2.6 ± 0.13 μm, porosity 7.6 ± 1.4%, average pore size 1.44 ± 0.17 μm; nano-Ti/PEO: *R_a_* = 2.5 ± 0.05 μm, porosity 7.6 ± 1.5%, average pore size 0.61 ± 0.11 μm	c(RGDfC), cPGD-PH	-	Cell viability: FLECH-104, MSC, MG-63. c(RGDfC) reduces cell viability due to the toxicity. The appearance of a linker and a bisphosphonate anchor reduced the toxicity of cyclo-RGD, ε-aminocaproic acid derivative with an SMCC linker increased the degree of fibroblasts and MSC cell proliferation on the CG-Ti/PEO and nano-Ti/PEO.	[84]
Ti	Na_3_PO_4_·12H_2_O, pulsed bipolar mode, positive pulse: voltage 470 V, duty cycle 51%, negative pulse: voltage 40 V, duty cycle 26%, frequency 300 Hz, 20 °C, t = 5 min	-	HYA–LL37	-	Antibacterial efficacy: *S. aureus*, *P. aeruginosa*, *E. faecium*, *E. coli*. PEO/HYA–LL37 demonstrated a significant (*p* < 0.05) suppression of the ability of bacteria to form biofilms.	[85]
